# Deciphering intra-connectivity of gene network response to drought and salinity in apple

**DOI:** 10.3389/fpls.2026.1763760

**Published:** 2026-03-16

**Authors:** Ke Li, Jirong Zhao, Zhikun Li, Wenxuan Chu, Ruigang Wu, Zhenyu Huang

**Affiliations:** 1 School of Landscape and Ecological Engineering, Hebei University of Engineering, Handan, Hebei, China; 2 Shaanxi Key Laboratory of Research and Utilization of Resource Plants on the Loess Plateau, College of Life Sciences, Yan’an University, Yan’an, Shaanxi, China; 3 Zhongyuan Research Center, Chinese Academy of Agricultural Sciences, Xinxiang, China; 4 Zhengzhou Fruit Research Institute, Chinese Academy of Agricultural Sciences, Zhengzhou, China; 5 Chuxiong Yunguo Agriculture Technology Research Institute, Chuxiong, China

**Keywords:** apple, hormone signal transduction, MdSNF1, salt-drought stress, transcriptome analysis

## Abstract

Drought and salt stresses severely constrain apple yield and quality. Using *in vitro* plantlets of apple ‘*Golden Delicious*’ as experimental materials, we aimed to dissect the stress-responsive mechanisms and provide a basis for the genetic improvement of apple stress resistance. Plantlets under NaCl (salt stress) and PEG (drought stress) treatments, with samples collected at 0, 1, 6, 12 and 24 h post-treatment for transcriptome sequencing. Gene expression levels were quantified as FPKM values to analyze temporal dynamic changes, and Differentially Expressed Genes (DEGs) were screened with |log2(FoldChange)| >1 and padj<0.05. Co-expression analysis was performed to explore the interaction patterns of stress-related genes, coupled with Gene Ontology (GO) (p-value<0.05) and Kyoto Encyclopedia of Genes and Genomes (KEGG) (FDR<0.05) enrichment analyses. The core regulatory network of the “hormone signal-metabolic pathway” in apple in response to salt/drought stresses was identified, with the key nodes of this network clarified as *MdAREB3/MdJAZ1*, *MdPETE1*, *MdSNF1* and *MdGH9C2*, which are involved in hormone signal transduction, photosynthesis and carbohydrate metabolism, respectively. Stage-specific expression differences of key DEGs at 0, 1, 6, 12 and 24 h post NaCl/PEG treatments were further characterized via comparison of their expression patterns under the two stress conditions. This study systematically identifies the core regulatory network and its key nodes in apple in response to salt and drought stresses, thereby providing a reliable molecular basis for deciphering stress resistance mechanisms, verifying key gene functions, and the genetic improvement of salt/drought-tolerant apple varieties.

## Introduction

Apple (*Malus × domestica* Borkh.), as a temperate fruit tree widely cultivated worldwide, occupies a pivotal position in fruit production and agricultural economy (Fang et al., 2023). However, abiotic stresses such as drought and salinization have become bottlenecks constraining the sustainable development of the apple industry against the backdrop of intensifying climate change and increasing strain on arable land resources (Zhang et al., 2024; Yang et al., 2019a). Approximately, 40.0% of the world’s arable land is affected by drought to varying degrees, and saline areas are expanding at a rate of 1.0×10^6^~1.5×10^6^ hm² per year. Drought and soil salinization seriously affect the physiological and metabolic processes of apples, resulting in economic losses (Verma et al., 2024).

As a crucial component in apple cultivation, the growth and development status of apples, along with their stress tolerance, directly determine the fruit yield and quality of the scion varieties (Li et al., 2023). Superiors can effectively mitigate abiotic stress damage to the entire plant by regulating their own physiological metabolism and molecular mechanisms. For instance, they resist drought and salt stress by enhancing root water and nutrient uptake capacity, accumulating osmotic regulators, and activating antioxidant system (Mao et al., 2022; Kong et al., 2017; Balfagón et al., 2022). Therefore, conducting an in-depth analysis of the molecular regulatory networks governing apple responses to drought and salt stress, and identifying key functional genes involved in stress signal transduction, holds significant theoretical value and practical significance for breeding new apple varieties with enhanced stress tolerance and improving the resilience of the apple industry.

Drought and salinity-increasingly severe environmental stresses-constrain plant growth, limit agricultural productivity, and threaten global food security. As a core stress, soil salinity impairs plant performance via inducing cellular osmotic stress, disrupting ion homeostasis, and triggering excessive reactive oxygen species (ROS) accumulation, which causes intracellular oxidative damage and compromises plant physiological metabolism (Wang and Chang, 2024; Zhou et al., 2024; Ikram et al., 2025). Drought and salt stresses exhibit unique and overlapping signals. For instance, the phosphatidylethanolamine-binding proteins CaMFT02 and CaMFT03 in pepper exert unique roles in response to drought and salt stresses (Lim et al., 2023). The primary signal triggered by drought is osmotic stress, while salt stress exerts both osmotic stress and ionic stress (or ionic toxicity) effects on cells (Osku et al., 2025; Islam et al., 2025; Chen et al., 2022; Owoyemi et al., 2025). The secondary effects of drought and salt stress are complex, involving damage to cellular components such as oxidative stress, carbohydrate metabolism, membrane lipids, proteins, and nucleic acids. Plant cells employ various mechanisms to withstand and reverse the cellular effects of drought and salt stress, thereby balancing oxidative stress (Ma et al., 2020; Haghpanah et al., 2024; Ahanger et al., 2021; Wang and Chang, 2024; Zhou et al., 2024; Ikram et al., 2025).

With the rapid advancement of high-throughput sequencing technologies, transcriptomics has emerged as a crucial tool for investigating the molecular mechanisms underlying plant responses to abiotic stresses. Currently, relevant transcription factor (TF) families, such as AP2-EFR, Dof, MADS-box, bZIP, CPP, ZF-HD, and GATA, have been reported and identified in cowpea under drought stress (Ferreira-Neto et al., 2021). Transcriptomic analysis has been employed to discuss the identification of TFs in pepper and the pathways involved in drought stress tolerance. Notably, within the *AP2/ERF* family, dehydration-responsive element-binding factors (DREB) and CRT element-binding factors have been extensively characterized: their roles in protein structural stability, DNA binding, and post-translational modification have been well studied through transgenic research (Negi et al., 2021). A transcriptomic study conducted by Aleem et al. (2021) demonstrated significant differences in gene expression patterns between sensitive crop cultivars and tolerant crop cultivars. Additional genes induced during drought include those encoding osmolytes (e.g., proline, glycine betaine, and sugars), as well as genes involved in abscisic acid (ABA) biosynthesis pathways, signaling proteins, antioxidant components, and TFs. In terms of salt stress, in *Arabidopsis*, under salt stress conditions, *Arabidopsis* lines with increased *Asg2* expression exhibit inhibited primary root elongation, reduced seed germination rate, and enhanced sensitivity of leaves and seedlings to salt stress. *CdWRKY50* functions as a negative regulator that mediates the response of bermudagrass to salt stress (Tian et al., 2024a; Huang et al., 2021). In *rice* (*Oryza sativa*), *osSCYL2* is involved in phytosterol accumulation and regulates plant growth as well as the response to salt stress (Xu et al., 2024). Thus, transcriptomic technologies have been utilized to elucidate the molecular mechanisms underlying the responses of model plants (such as *Arabidopsis*, *rice*, and *maize*) and some fruit crops to drought and salt stresses, leading to the identification of a set of stress-responsive signaling pathways and associated genes (Ahmad et al., 2019; Tian et al., 2024b; Yu et al., 2020a). Among these, endogenous plant hormones—including auxin (indole-3-acetic acid, IAA), abscisic acid (ABA), gibberellin A3 (GA3), and brassinosteroids (BRs)-play crucial roles in mediating the responses of higher plants to stresses (Li et al., 2019). Additionally, genes related to abscisic acid (ABA) biosynthesis (e.g., NCED), TF genes (e.g., *AKR*, *NAC*, *ZIP*, *WRKY*, and *MYB*), and antioxidant enzyme genes have been identified to be involved in mediating the response mechanisms to drought and salt stresses (Changan et al., 2023; Yu et al., 2020b; Pan et al., 2017; Zhu et al., 2018; Hao et al., 2024; Chanwala et al., 2024).

Apple (*Malus domestica.*), one of the most widely produced and economically important fruit crops in temperate regions. During the long-term domestication process, the quality and yield of cultivated apples are greatly improved, but their global cultivation and promotion are limited by drought and high salt stress. So how apple plants sense stress signals and adapt to adverse environments are fundamental biological questions. In recent years, there have been some researches about the regulating mechanism of apple drought and salt tolerance. In apple, *MdMYB46* could enhance the salt and osmotic stress tolerance not only by activating secondary cell wall biosynthesis pathways but also by directly activating stress-response signals (Chen et al., 2019). The previous study conducted a series experiments verifying that the *MdNAC047* gene was significantly induced by salt treatment and found a novel “*MdNAC047-MdERF3-*ethylene-salt tolerance” regulatory pathway, which provides new insight into the link between ethylene and salt stress (An et al., 2018). In the apple, 38 *FKBP* genes were identified, and found that the pairing in the *MdFKBP62a/MdFKBP65a/b*-mediated network is involved in water-deficit and salt-stress signaling, both of which are uniformly up-regulated through interactions with heat shock proteins in apple (Dong et al., 2018). *MdcyMDH* enhances the tolerance of the transgenic plants to cold and salt modifying the redox signal and improving the cell reducing power, which promoting the interaction of redox and salicylic acid (Wang et al., 2016). The dynamic complexity of drought and salt stress control network of apple increases the difficulty of systematic research (Verma et al., 2024), while second-generation sequencing (SGS, or called next-generation sequencing) provides a precise and comprehensive analysis of RNA transcripts for gene expression, and become a common tool to explore biological questions systematically (Thudi et al., 2012). By RNA-seq analysis of apple peels form the ‘*Red Fuji*’ cultivar, *MLNC3.2* and *MLNC4.6* function as eTMs for *miR156a* and prevent cleavage of *SPL2*-like and *SPL33* by *miR156a* during light-induced anthocyanin biosynthesis, providing fundamental insights into lncRNA involvement in the anthocyanin biosynthetic pathway in apple fruit (Yang et al., 2019b). RNA-seq analysis of 6-BA-treated ‘*Nagafu No.2*’ apple buds revealed that the up-regulation of cytokinin (CK) signal components and gibberellin (GA) signal repressors contributes to the promotion of floral transition; this finding provides insights into the responses of flowering- and development-related pathways, as well as key TFs (i.e., *SPLs*, *SOC1*, *FD*, and *CO*L), to 6-BA during *apple* floral transition (Li et al., 2019). Moreover, dopamine may affect apple drought tolerance by regulating the expression of *WRKY*, *ER*F and *NAC* TFs, activating the expression of *CNGC* and *CAM/CML* family genes to improve drought tolerance (Gao et al., 2020). However, due to significant differences in genetic backgrounds and stress resistance mechanisms among different plant species, the unique molecular regulatory mechanisms underlying the response of apple s to drought and salt stresses still require further in-depth investigation.

In current study, apple trees were treated with NaCl and PEG to simulate salt and drought stress conditions, respectively. Dynamic changes in the stress response were monitored over the treatment period. Via time-series RNA sequencing, we explored the expression profiles of key genes involved in the drought and salt stress response mechanisms, as well as the intrinsic connections between the regulatory mechanisms underlying these two distinct stress responses. Furthermore, through transcriptomic analysis, a set of candidate genes that participate in regulating apple responses to drought and salt stresses. This work lays a foundation for the subsequent functional exploitation of these genes to develop apple cultivars with desired levels of drought and salt tolerance.

## Materials and methods

### Plant materials

#### RNA preparation for RNA-seq

Micro-propagated *‘Golden Delicious’* apple plantlets were grown in tissue culture at Hebei University of Engineering, Handan, China. The cuttings were maintained under a 16 h light at 25 ± 1°C, followed by 8 h dark at 15 ± 1 °C. The stem cuttings were divided into two groups. The first group of cuttings was treated with NaCl to simulate salt stress. The rooting medium was composed of 1/2 MS, 200 mmol·L^-1^ Nacl, 25 g·L^-1^ sugar, 7.5 g·L^-1^ agar, and pH 5.8; The concentration of 200 mmol·L^-1^ NaCl was finally determined after screening by gradient concentration pre-experiments and referring to the commonly used effective stress concentrations in recent similar apple salt stress studies (Zhang et al., 2020; Yang et al., 2009). The second group of cuttings was treated with PEG, the medium was composed of 1/2 MS, 20.0% PEG (4000), 25 g·L^-1^ sugar, 7.5 g·L^-1^ agar, and pH 5.8; similarly, the 20.0% PEG (4000) concentration was selected based on gradient concentration pre-experiments combined with the commonly used effective concentrations in recent apple drought stress studies (Zhang et al., 2012; Li et al., 2021). Both concentrations could stably induce stress responses in apple plantlets while avoiding excessive lethal damage to the experimental materials. Stem bark from 0.5–1 cm basal sections of 30 cuttings was frozen in liquid nitrogen at 0, 6, 12, 24, and 48 h after treatment for RNA-seq analysis.The collected samples were immediately immersed in liquid nitrogen and stored at −80 °C until used for further processing. Total RNA was extracted by the modified CTAB method (Added 2.0% (w/v) PVP-40 and 2.0% (v/v) β-mercaptoethanol to the CTAB extraction buffer to suppress polyphenol oxidation and RNA degradation—a critical optimization for apple stem bark samples with high polyphenol and polysaccharide content; adjusted the concentration of CTAB in the extraction buffer to 1.5% (w/v) and optimized the water bath conditions to 65°C for 30 min, enhancing the lysis efficiency of plant cells while minimizing RNA hydrolysis. Supplemented a two-step 75.0% ethanol washing process after RNA precipitation to remove residual salts and impurities, ensuring the purity of the extracted RNA) (Li et al., 2018). RNA quality was checked on 0.8% agarose gel and Nano Photometer Spectrophotometer (Implant USA), and the samples, which passed the quality tests (OD260/280 = 1.8~2.0), were chosen for RNA-seq analysis.

#### RNA-seq library construction, sequencing

For each sample, 5 μg total RNA was used to isolate mRNA to prepare an RNA-seq library using NEBNext Poly(A) mRNA Magnetic Isolation Module and NEBNext Ultra Directional RNA Library Prep Kit for Illumina (New England Biolabs, Ipswich, MA) following the manufacturer’s protocols. Specifically, 3 biological replicates were set for each group, including the control group (0 h post-treatment) and each of the 4 time points (1, 6, 12, 24 h post-treatment) under both NaCl and PEG stress treatments. The cDNA library was sequenced from both of 5’ and 3’ ends on the Illumina Hiseq2500 platform according to the manufacturer’s instructions, in which 150 bp paired-end reads were obtained. In total, 27 samples were sequenced, which was consistent with the experimental design (3 biological replicates per group × 9 groups: 1 control group, 4 time points under NaCl stress, and 4 time points under PEG stress).

#### RNA deep sequencing and sequencing data filtering

To ensure the quality of data, remove reads with adapters and containing N (N means that the base information cannot be determined) and the low-quality reads (reads whose base number of Qphred ≤ 20 accounts for more than 50.0% of the entire read length). After filtering the original data, checking the sequencing error rate, and the GC content distribution, clean reads for subsequent analysis are obtained. This project has sequenced 27 samples, and the average clean data of each sample is not less than 6.5 Gb.

### Reference genome alignment

Sequencing fragments were obtained by random fragmentation of mRNA. To determine genes that are transcribed from these fragments, use HISAT2 software to quickly and accurately compare clean reads with the reference genome (https://www.rosaceae.org/species/malus/malus_x_domestica/genome_GDDH13_v1.1), and obtain the positioning information of reads on the reference genome (Mortazavi et al., 2008).

### Gene expression abundance statistics

According to the position information of the gene comparison on the reference genome, the number of reads covered by each gene (including the new predicted gene) from the start to the end is counted. Filter out reads with a comparison quality value lower than 10, reads on unpaired comparisons, and reads that are aligned to multiple regions of the genome. This part of the analysis uses the feature counts tool in the sub-read software (Yang et al., 2014). The gene expression value of RNA-seq is generally not expressed by reading count but FPKM. FPKM expected the number of fragments per kilobase of transcript sequence per millions of base pairs sequenced. The FPKM values can reflect the gene expression. It has corrected the sequencing depth and gene length successively (Bray et al., 2016). In the current study, FPKM ≥ 1 was used as a standard for identifying gene expression. This standard filtered out the weakly expressed genes.

### Transcripts assembly and expression analysis

StringTie was used for transcript assembly and quantification for each RNA-Seq sample. DESeq2 software was used to detect differentially expressed genes (Love et al., 2014). The R package VennDiagram was used to generate the Veen diagram (Lam et al., 2016).

### Principal component analysis

To identify the replicates that have similar expression patterns, we performed PCA among all the samples. The raw count data were transformed in Deseq2 (Love et al., 2014) and then the principal component analysis was conducted using the princomp function from R. rgl 3 package form CRAN, was used to realize 3-D visualization.

### Differentially expressed genes

The documented transcripts within the reference genomic annotation file were analyzed by DESeq2 software for differential expression genes analyses (Love et al., 2014). Screening criteria of DEGs were |log_2_(FoldChange)| >1 and padj < 0.05. WGCNA (Weighted Gene Co-expression Network Analysis) was performed by previous research (Langfelder and Horvath., 2008). Additionally, two strategies were used to analyze the DEGs. The first was Gene Ontology (GO) functional enrichment and the second was pathway enrichment. GO enrichment analysis of functional significance applied a Fisher’s Exact Test to map all DEGs to the GO database terms. TopGO (version 2.18.0) software was used for the GO enrichment analysis (Alexa et al., 2013). The p-value was corrected by the Bonferroni test, and a corrected p-value < 0.05 was chosen as the threshold to define a significantly enriched GO term. KOBAS (kobas2.0-20150126) software was used for Kyoto Encyclopedia of Genes and Genomes (KEGG) Pathway enrichment analysis and statistical analyses were performed with the Hyper-geometric test (Chen et al., 2011).

### Identification of co-expression modules

The R package WGCNA (Langfelder and Horvath, 2008) was used to identify modules of highly correlated genes based on FPKM data. First, the R package DCGL (Differentially Coexpressed Genes and Links) filtered genes by expression and variation, retaining 14,293 genes (Nath et al., 2020). Using the pickSoftThreshold function in WGCNA, the soft thresholding power was set to 21 (interpreted as the correlation matrix soft threshold). The resulting adjacency matrix was converted to a topological overlap (TO) matrix via the TOM similarity algorithm. Genes were hierarchically clustered based on TO similarity, and the Dynamic Hybrid Tree Cut algorithm (30) was used to cut the clustering tree and define modules as tree branches. Each module’s expression profile was summarized by its first principal component (module eigengene), and modules with highly correlated eigengenes (r > 0.8) were merged. Genes in each module were then analyzed as described in “GO Enrichment Analysis and Pathway Enrichment Analysis”.

### Visualization of hub genes

Genes with the highest degree of connectivity within a module are referred to as intramodular hub genes (Langfelder and Horvath, 2008), the top 100 hub genes from each module from two conditions, ranked by KME, were selected. The hub genes in each module were compared. The top 100 hub genes of focused module CKM6 was visualized by VisANT, in the network, the genes belong only to CKM6 were marked green, and the hub genes both belong to CKM6 and A04M3 were marked red. And the gene annotation information was from KOBAS 2.0 annotation result.

## Results

### The transcriptomic profiles of apple seedlings under different treatments of NaCl and PEG

**Figure 1:**
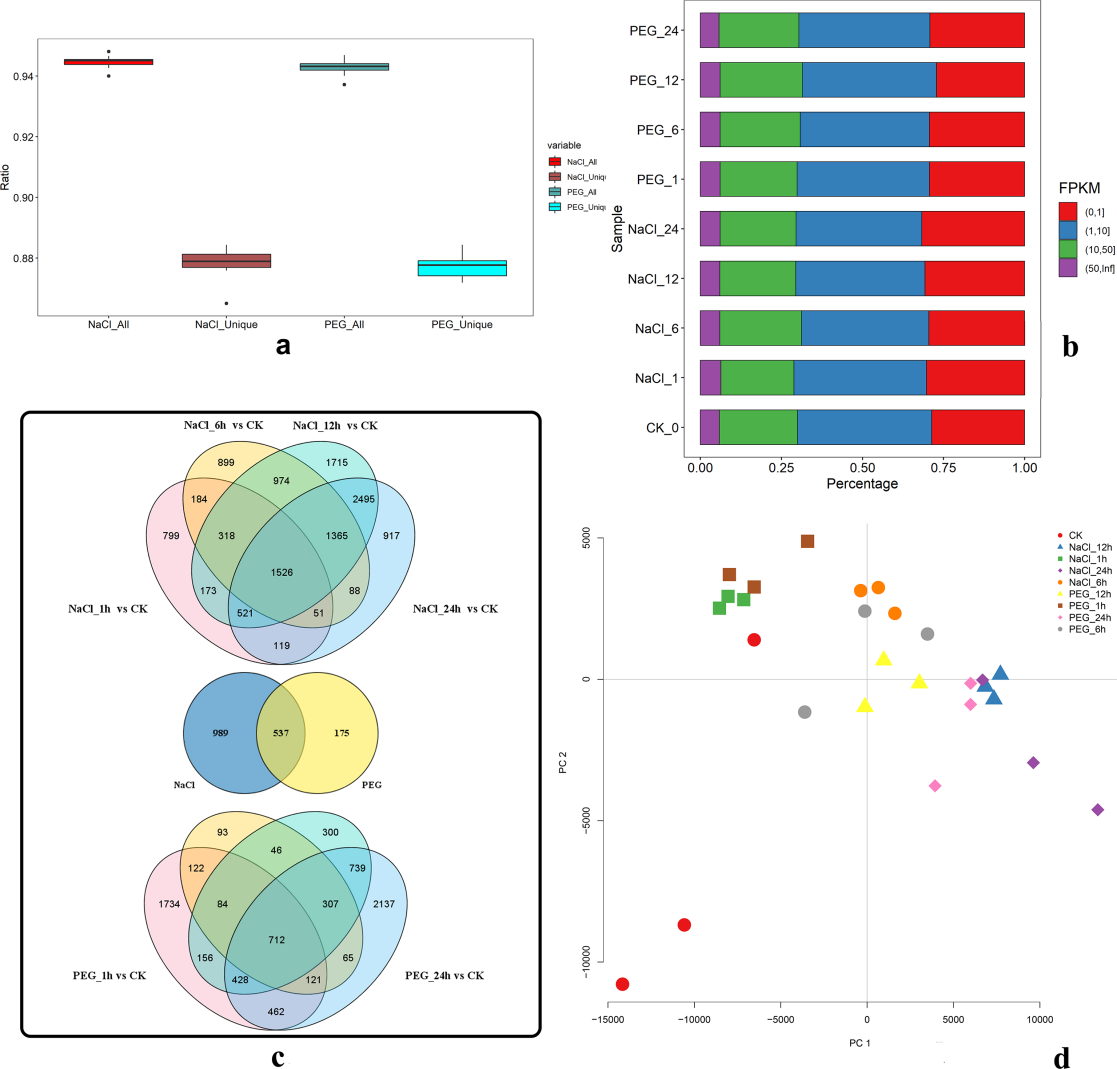
Transcriptomic profiling of key genes in apple s under NaCl (salt) and PEG (drought) stresses. **(A)** Box plots of Ratio distributions for all vs. treatment-unique genes under NaCl/PEG. **(B)** Stacked bars of FPKM proportions across gene categories in CK and NaCl/PEG (1/6/12/24 h) samples. **(C)** Venn diagrams of DEGs: (top) NaCl (1/6/12/24 h) vs. CK; (middle) NaCl- vs. PEG-specific DEGs; (bottom) PEG (1/6/12/24 h) vs. CK (numbers = DEG counts). **(D)** PCA plot (FPKM-based) clustering CK and NaCl/PEG (1/6/12/24 h) samples by PC1/PC2.

To investigate the transcriptome dynamics of apple seedlings’ response to salt and drought stress, we collected the samples from seedlings under the treatment of NaCl and PEG at 0, 1, 6, 12, and 24 h, constructed 27 libraries, and performed time-series RNA-sequencing (RNA-seq) experiment. We obtained 475.5 million high-quality paired-end reads (average ~17.6 million for each sample) after removing low-quality reads and adaptors, of which 94.4% was aligned to the apple DH genome (**Figure 1A**; **Supplementary Table 1**). All the genes were quantified and assigned with the fragments per kilobase of transcript per million mapped reads (FPKM). 42,916 genes were detected to be expressed across all the samples, of which more than 18784 genes (~40.0%) genes had low expression levels (FPKM ≤1), and nearly 12,041 (~28.0%) genes had higher expression levels (FPKM >10) (**Figure 1B**; **Supplementary Table 2**). Based on the expressive matrix, Pearson correlation analysis and hierarchical cluster analysis revealed a good correlation between biological replicates (**Figure 1D**; **Supplementary Figure 1**). Parallelly, FPKMs of three randomly selected genes exhibited a high Pearson correlation efficiency (r^2^ = 0.84, *P*-value <0.01) (**Supplementary Figure 1**).

By pairwise comparisons of the 27 libraries, 18,707 differentially expressed genes (DEGs) were identified. During NaCl treatment groups, the number of most DEGs compared between NaCl_1 h and NaCl_24 h was 790, while in PEG treatment groups, that was 1336 detected in the comparison between PEG_1 h and PEG_24 h (**Supplementary Table 3**, **Supplementary Figure 2**).

Setting the time-series experiments was to discover the major and possibly shared regulatory networks of apples under drought stress (PEG treated) and salt stress (NaCl treated). Thence, the DEGs between different treatments and controls were mainly analyzed. Compared with control, a total of 12,144 and 7,506 differentially expressed genes (DEGs) were detected under NaCl and PEG treatments, respectively. Among it, 1526 and 712 DEGs were shared across four corresponding treatments respectively, and 531 DEGs overlapped between NaCl and PEG treatments (**Figure 1C**; **Supplementary Table 3** and **Supplementary Figure 3**).

Additionally, under salt stress, there were 542 up-regulated genes, including a large number of redox-related genes, carbohydrate metabolism, protein and lipid metabolism-related genes, secondary metabolism genes, cell wall degradation synthesis related genes, osmotic regulation ionophore genes, and hormone transduction regulation genes represented by abscisic acid. Correspondingly, in 929 down-regulated genes, there were a series of hydrolase coding genes, reverse regulated abscisic acid transduction genes, pectin methyl-esterase inhibitor genes, transmembrane ion transporter genes, and photosystem (plant photosynthetic related) coding protein genes (**Supplementary Figure 3**, **Supplementary Table 3**).

Similarly, under PEG treatment, 186 up-regulated DEGs mainly concentrated on redox genes, hormone signal transduction genes, and calcium ion signal transduction genes. In contrast, among 514 down-regulated genes, there were a large number of redox-related genes, membrane transport-related genes, proline-rich protein-coding genes, cell wall synthesis related genes, and photosystem (plant photosynthetic related) protein-coding genes (**Supplementary Figure 3**, **Supplementary Table 3**). DEGs with functions of different biological processes, such as signal transduction and cell wall degradation synthesis, showed similar expression patterns under salt stress and drought stress. Furthermore, the DEGs that showed a common expression pattern under the NaCl and PEG treatments were 152 (up-regulated) and 379 (down-regulated), a total of 531, accounting for 98.9% of the overlapping 537 differential genes under all treatments (**Figure 1**; **Supplementary Figure 3**).

### Identification of conversed and divergent gene expression modules

**Figure 2:**
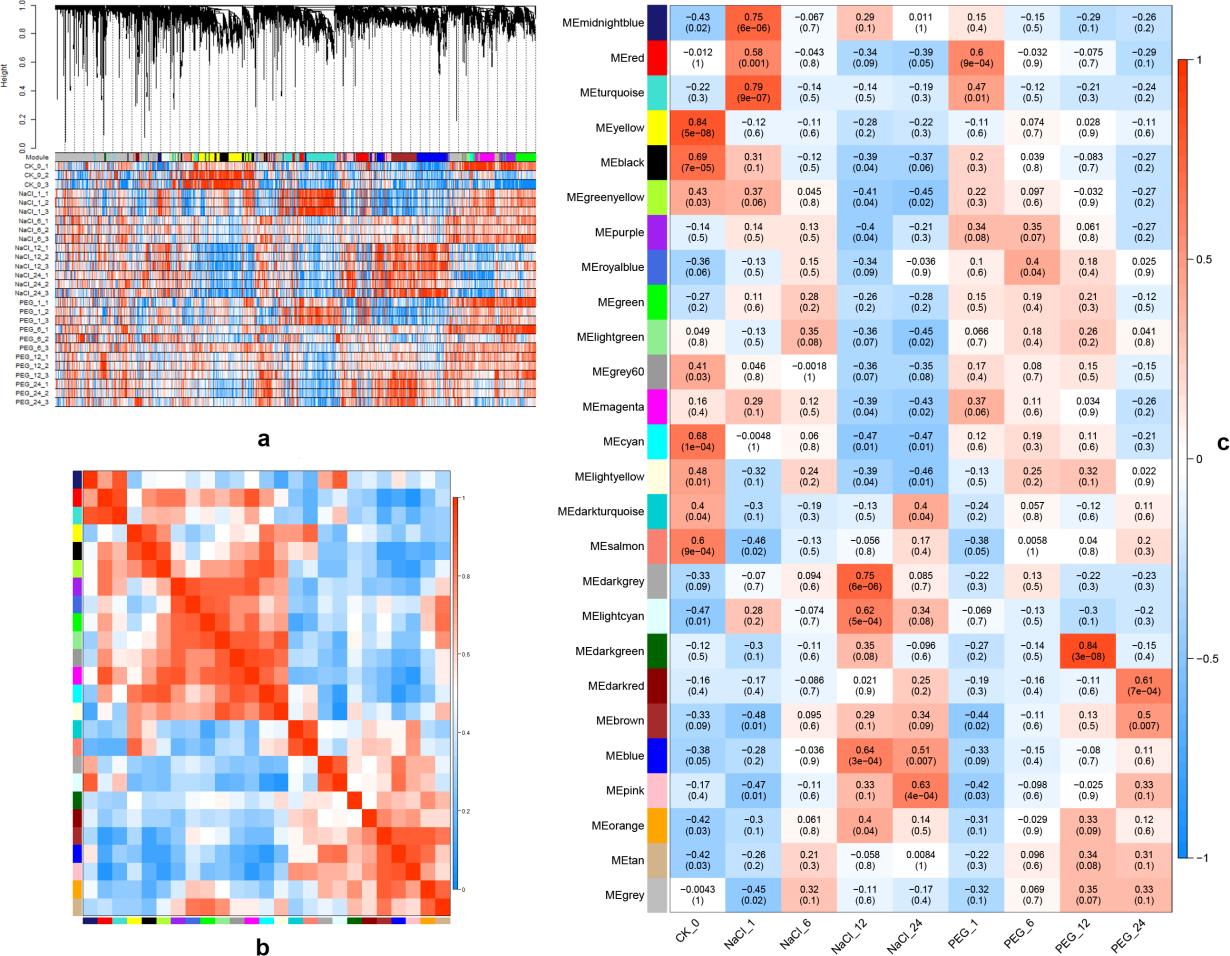
Co-expression module analysis of genes in apple s under NaCl/PEG treatments. **(A)** Hierarchical clustering tree (top) and gene module assignment (bottom). **(B)** Heatmap of gene expression across co-expression modules. **(C)** Module-sample correlation matrix (color, correlation; numbers, correlation/p-value) for CK/NaCl/PEG treatments.

To investigate the gene regulatory network (GRN) during the response of salt and drought treatments, the current study identified co-expressed gene sets via weighted gene co-expression network analysis (WGCNA). After filtering 46558 genes based on the expression (50.0% filtered) and variation (29.8% filtered) across all the samples via DCGL package, the 9,397 remaining genes fell into 26 co-expression modules, and the number of genes harbored in 25 co-expression modules (except the unassigned grey module containing 2,442 genes) ranged from 37 to 870 (**Figure 2**). Setting 0 and 1 as the phenotype of nine (control, 4 NaCl and 4 PEG) treatments, we conducted an association analysis between modules and phenotype to identify important modules and relative hub genes (**Figure 2**). Each module except MEgreen was associated with at least one treatment. Among them, the correlation coefficient of five modules were all above 0.75 (*p*-value < 0.01). A total of 13 modules were significantly related to control, 17 modules to NaCl time-series treatments, and 8 modules to PEG time-series treatments respectively. It is obvious that various genes distributed in more modules under NaCl treatments were more than PEG treatments, which correspond to DEGs analysis and may predict that when responding to salt tolerance, the biological process and plant inner genes regulatory networks were more complex than to drought tolerance. In addition, among the 25 modules, 13 modules were related to at least two treatments, 8 modules to three treatments (**Figure 2C**), and only one module MEsalmon (*p*-value < 0.05) was shared through control, NaCl (NaCl_1 h) and, PEG (PEG_1 h) treatments (**Figure 2C**).

Connecting with the database information, the TFs (Transcriptional factors) and TRs (Transcriptional receptors) distributed in the 25 modules were identified. Among the 49 TFs families, the number of the genes of WRKY, ERF, NAC, and MYB were in the top 5 and all these gene families had taken part in the regulatory network mechanism of the plant response to various stress reported by many types of research.

### Deciphering key co-expression modules

**Figure 3:**
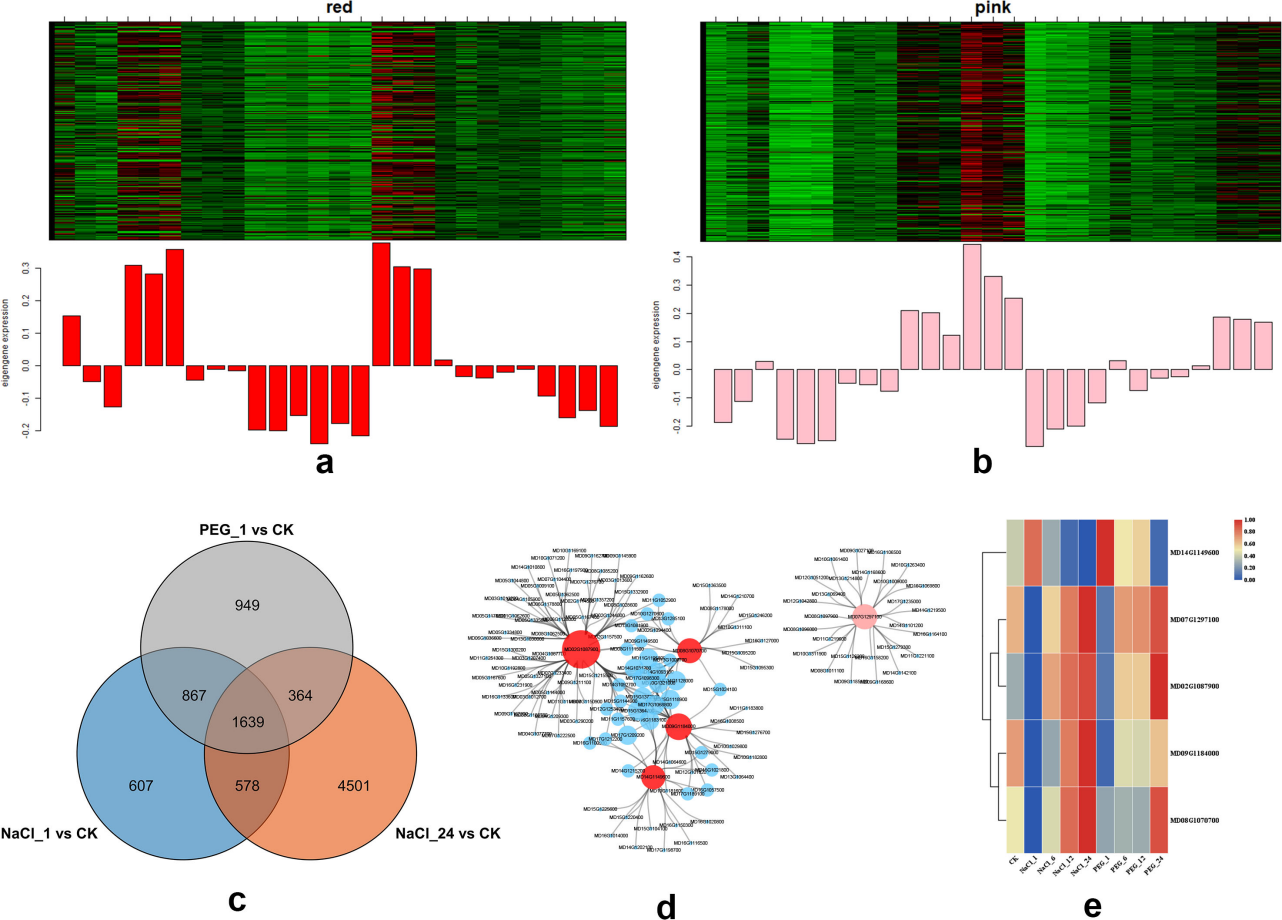
Gene analyses of key modules in apple s under NaCl/PEG treatmenets. **(A)** Heatmap (top) and expression profile (bottom) of the red module. **(B)** Heatmap (top) and expression profile (bottomcccvcidate genes. **(C)** Venn diagram showing the number of differentially expressed genes (DEGs) in apple under PEG_1 vs CK, NaCl_1 vs CK, and NaCl_24 vs CK treatments, and their overlaps. **(D)** Co-expression network of hub genes in the red and pink modules, with red nodes representing core hub genes and blue/pink nodes representing co-expressed genes. **(E)** Heatmap of the expression patterns of key hub genes from the red and pink modules across different treatments (CK, NaCl_1, NaCl_24, PEG_1, PEG_6, PEG_12, PEG_24).

After browsing the module-trait table, we found MEred and MEpink had completely opposite expression pattern and both significantly related to NaCl_1 h, NaCl_24 h and PEG_1 h treatments (**Figure 2C**). As shown in **Figure 3A**, a red module was identified by WGCNA, in which genes exhibited distinct expression patterns across different stress treatments. The module eigengene was significantly upregulated under PEG and NaCl treatments, implying that this module was involved in stress response. The number of DEGs overlapped in NaCl_1 h, NaCl_24 h, PEG_1 h (all compared with control) was 1639, which distributed in the module MEred and MEpink was 42, 35. Filtering hub genes in the two modules by setting KME≥0.85 (Epigengene-based connectivity), only remained in MEpink (29) and MEred (33), respectively. For example, there were only five genes belonging to TFs families (MD07G1297100, MD14G1149600, MD09G118400, MD02G1087900, MD08G1070700) (**Figure 3B**; **Supplementary Table 4**). *MdHB7* (MD07G1297100) encodes homeobox-leucine zipper protein HB7 and may act as growth regulators induced by abscisic acid in response to water deficit. The expression of *MdHB7* was both lower during earlier treating phases (1~6 h) than control, while at latter especially at 24 h treating that were significantly higher than control. **Figure 3C** (Venn diagram) revealed 4501, 949, and 607 differentially expressed genes (DEGs) in NaCl*24 vs CK, PEG*1 vs CK, and NaCl_1 vs CK, respectively, with 1639 DEGs shared among the three groups, representing a core set of stress-responsive genes. **Figure 3D** displayed the co-expression network of the red module, where hub genes (red nodes) and their highly co-expressed genes (blue nodes) were identified, providing key candidate regulators in stress response. Furthermore, the expression heatmap in **Figure 3E** validated that these hub genes showed specific expression profiles under various stress treatments, supporting their central roles in the regulatory network of stress responses. Based on the identified co-expression modules, we further explored the core functional pathways and biological significance of the genes within each module through GO and KEGG enrichment analyses.

### DEGs in RNA-sequencing were screened with GO and KEGG pathways analyzed

**Figure 4:**
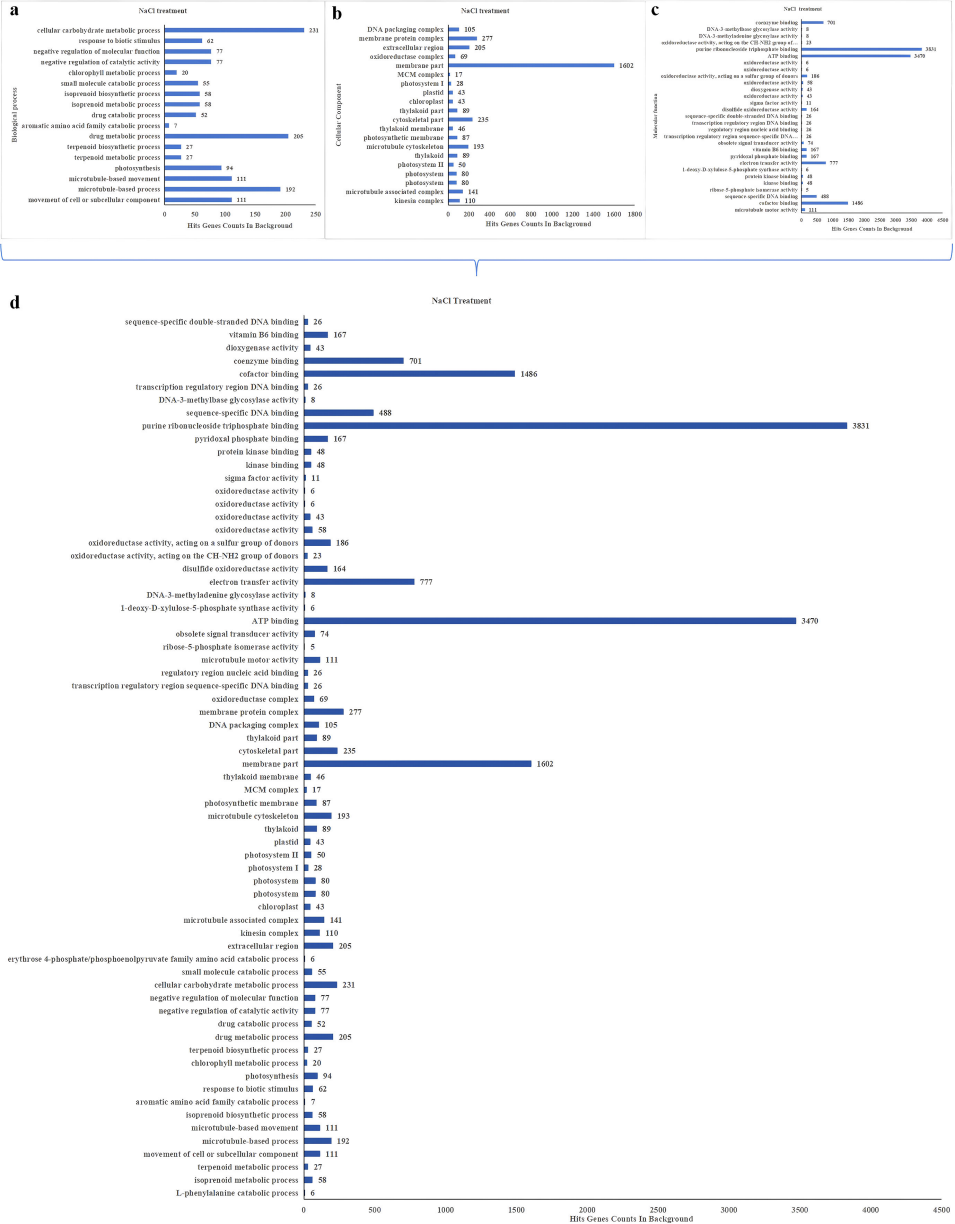
GO classification of DEGs under NaCl treatments. **(A–D)** Bar charts of GO enriched functional terms (y-axis) and their background gene counts (x-axis) across distinct functional categories, where subfigure **(A)** corresponds to biological process, **(B)** corresponds to cellular component, **(C)** corresponds to molecular function, and **(D)** is the summary of the three functional categories mentioned above.

**Figure 5:**
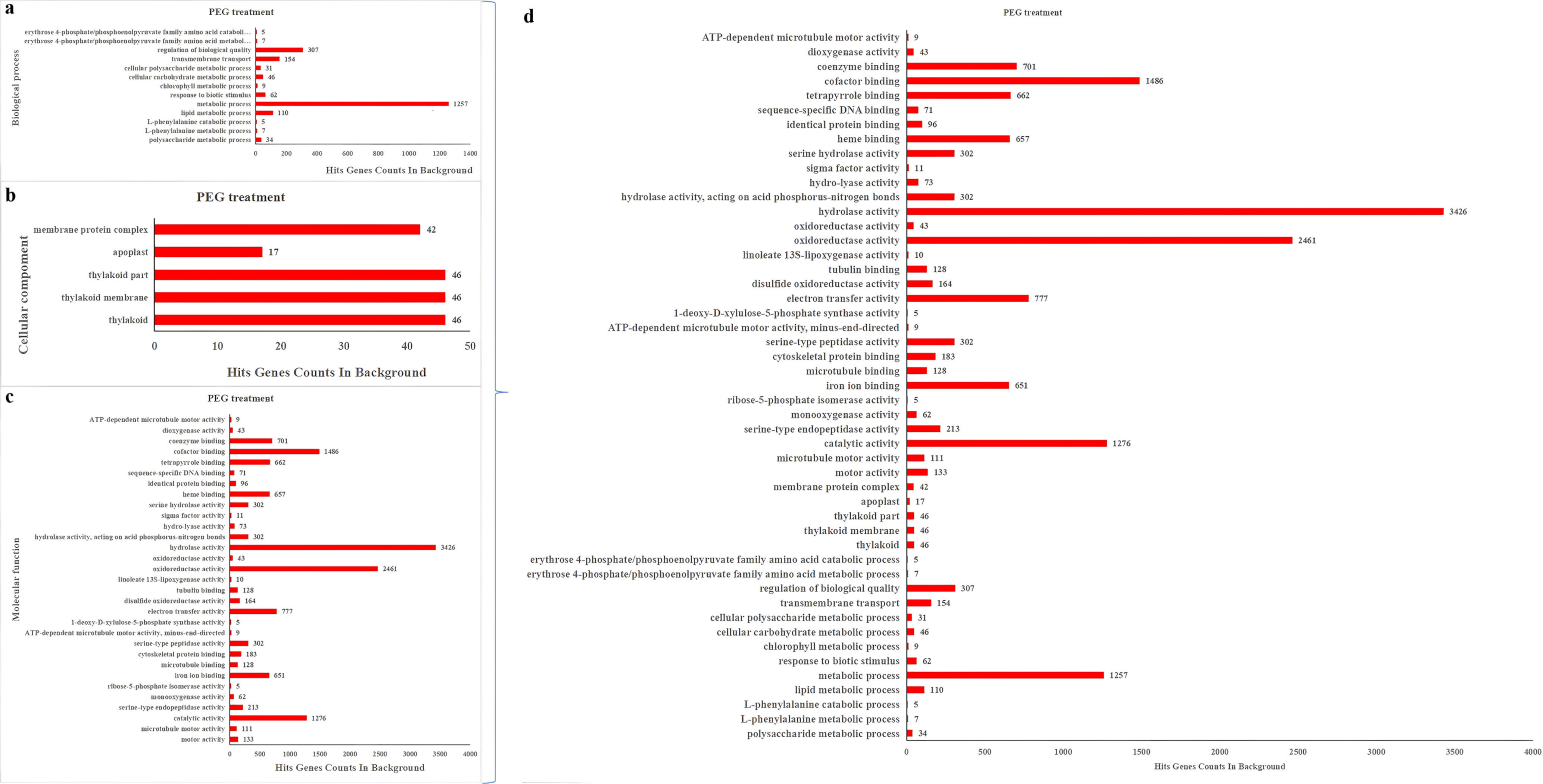
GO classification of DEGs under PEG treatments. **(A–D)** Bar charts of GO enriched functional terms (y-axis) and their background gene counts (x-axis) across distinct functional categories, where subfigure **(A)** represents biological process, **(B)** represents cellular component, **(C)** represents molecular function, and **(D)** summarizes the three GO functional categories.

The DEGs were also analyzed by GO enrichment. Significant enrichment pathways of DEGs were screened out at each time stage with NaCl and PEG treatments (**Figures 4A–C**, **5A–C**), and the final summary is presented in **Figures 4**, **5**. The Go analysis revealed that DEGs is associated with biological process, cellular component, and molecular function. Gene ontology (GO) enrichment analysis found that DEGs were distributed in 68 GO terms (corr p-value< 0.01) under NaCl treatments (**Figure 4D**), 49 GO terms (corr p-value< 0.01) under PEG treatment (**Figure 5D**), respectively. Among these modules, GO terms related to purine ribonucleoside triphosphate binding, ATP binding, membrane part, cofactor binding, and electron transfer activity were enriched across the four NaCl treatments (**Figure 4**), while GO terms associated with hydrolase activity, oxidoreductase activity, cofactor binding, catalytic activity, and metabolic process were enriched across the four PEG treatments (**Figure 5**).

**Figure 6:**
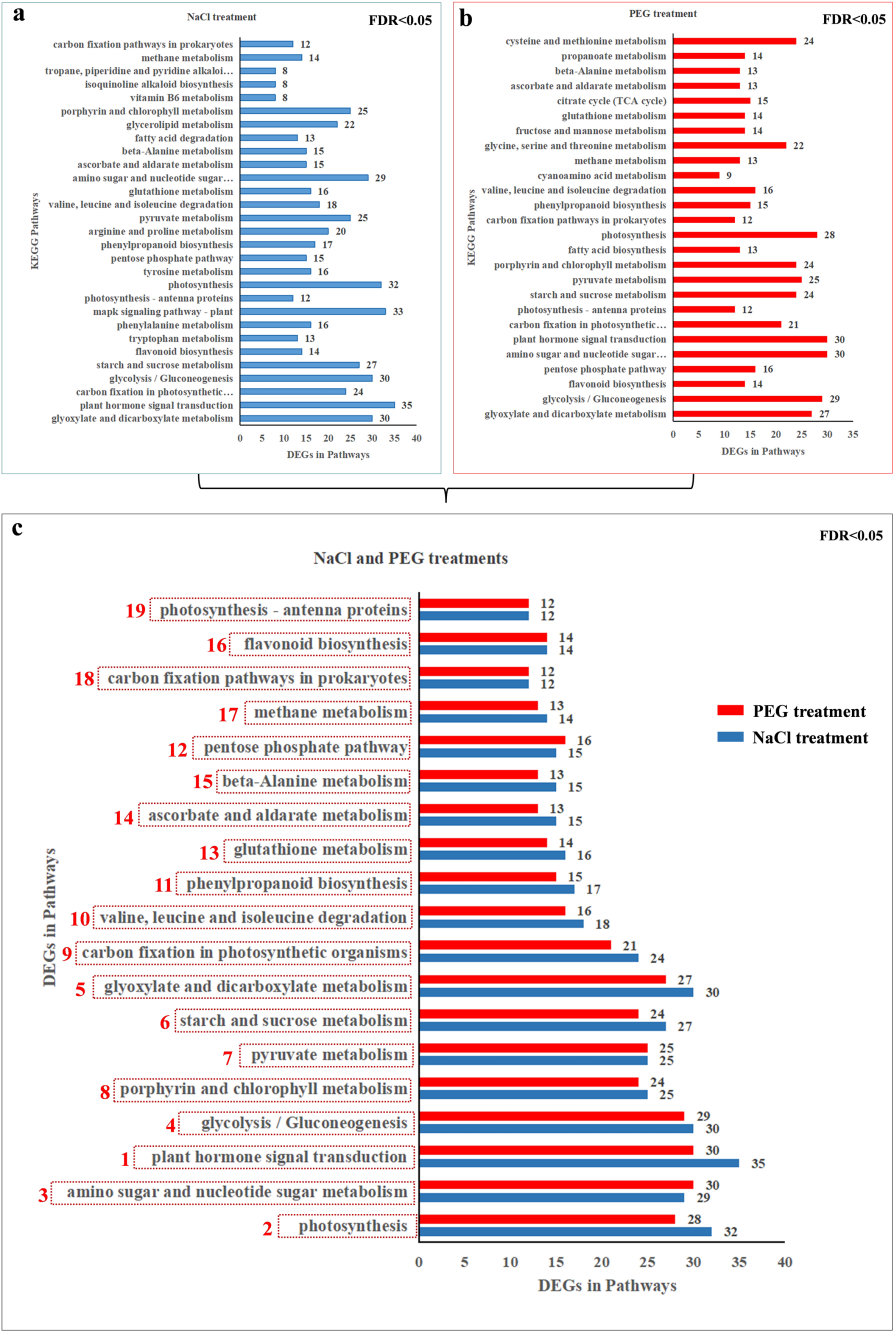
Deferentially Expressed Genes (DEGs) KEGG Pathway enrichment was analyzed in different stages (1, 6, 12, 24 h) under NaCl/PEG treatments (FDR<0.05). **(A)** Enriched pathways & DEG counts under NaCl treatment. **(B)** Enriched pathways & DEG counts under PEG treatment. **(C)** Shared enriched pathways (red=PEG; blue=NaCl) with respective DEG counts.

Similarly, significant enrichment pathways were also summarized by Kyoto Encyclopedia of Genes and Genomes (KEGG) enrichment pathway analysis (**Figure 6**). A total of 19 significant KEGG enrichment pathways were identified in **Figure 6C**. KEGG enrichment pathways analysis of DEGs revealed that 29 pathways (FDR<0.05) were enriched in NaCl treatments (**Figure 6A**), 26 pathways (FDR<0.05) were enriched in PEG treatments respectively (**Figure 6B**), and the overlap of them were 19 pathways (FDR<0.05) (**Figure 6C**).

**Figure 7:**
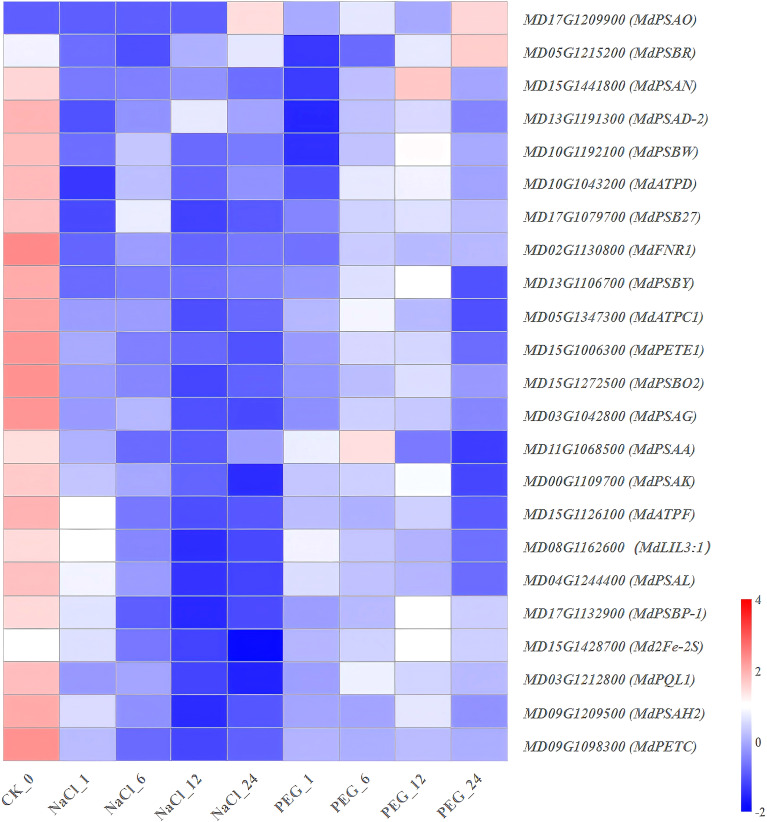
Heat map diagram of the log2 (FPKM) values for genes annotated as plant hormone signal transduction-related genes and root structure analysis under different treatment groups (control, NaCl, and PEG).

**Figure 8:**
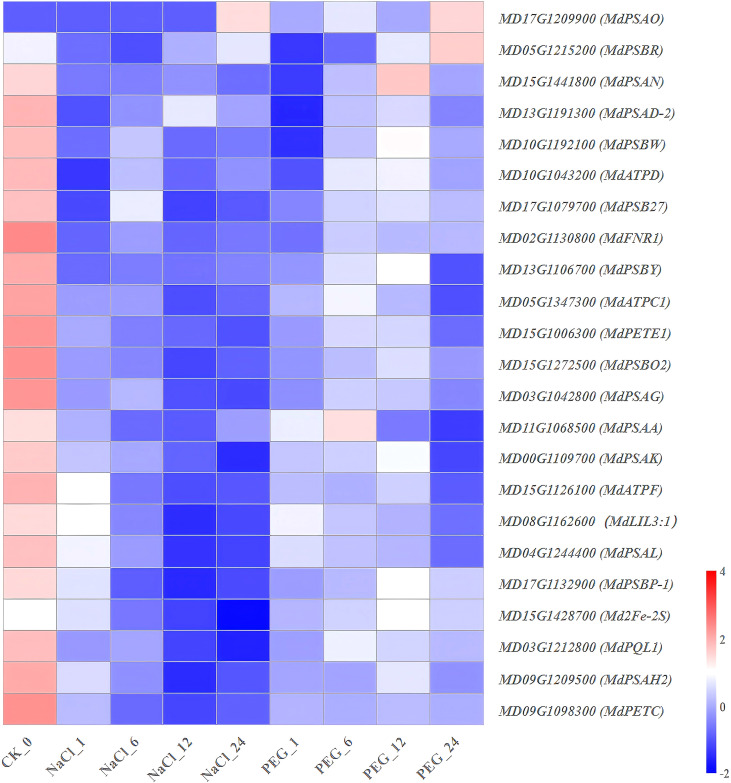
Heat map diagram of the log2 (FPKM) values for genes annotated as photosynthesis pathway-related genes and root structure analysis under different treatment groups (control, NaCl, and PEG).

**Figure 9:**
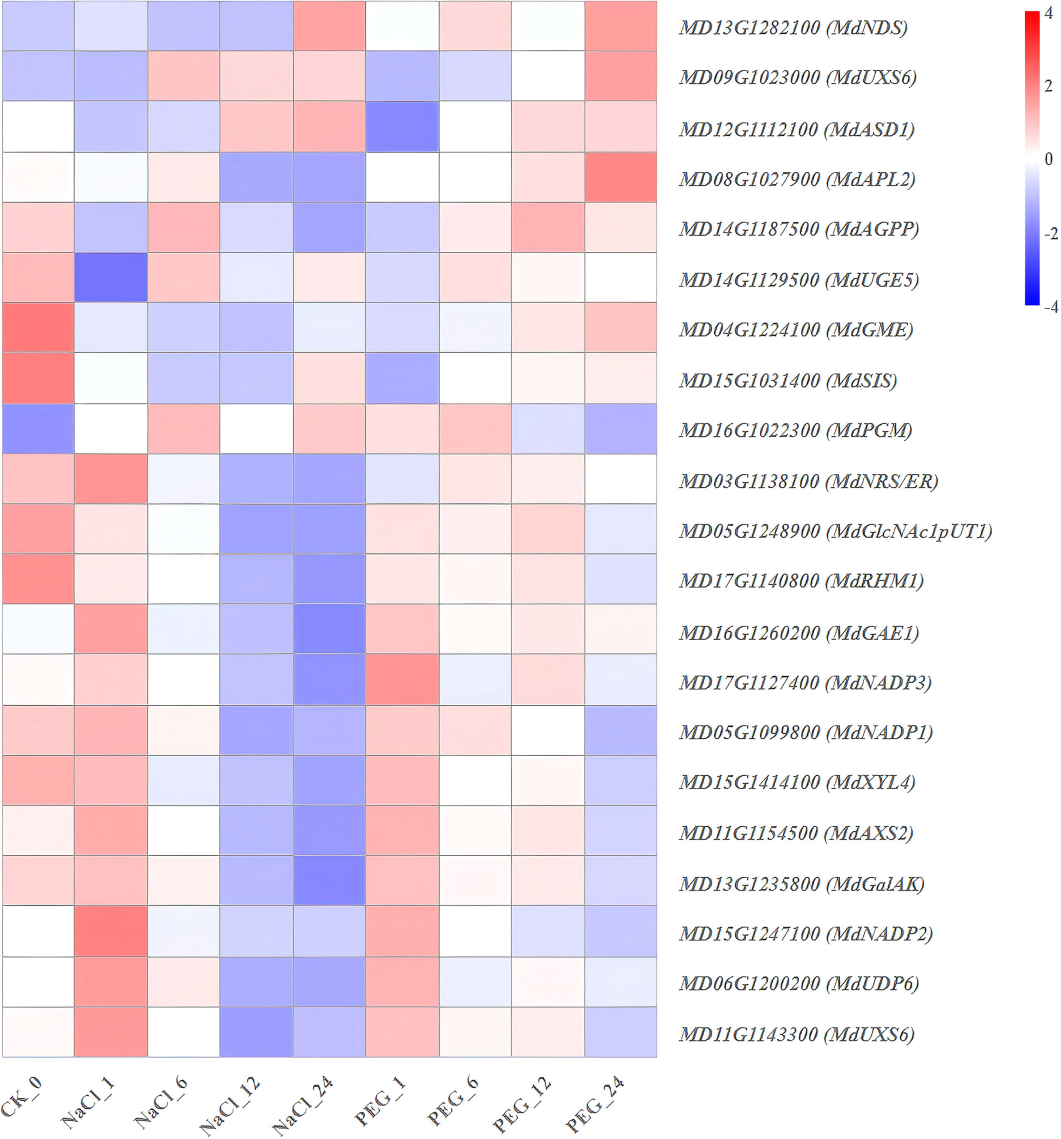
Heat map diagram of the log2 (FPKM) values for genes annotated as amino sugar and nucleotide sugar metabolism-pathway genes under different treatment groups (control, NaCl, and PEG).

**Figure 10:**
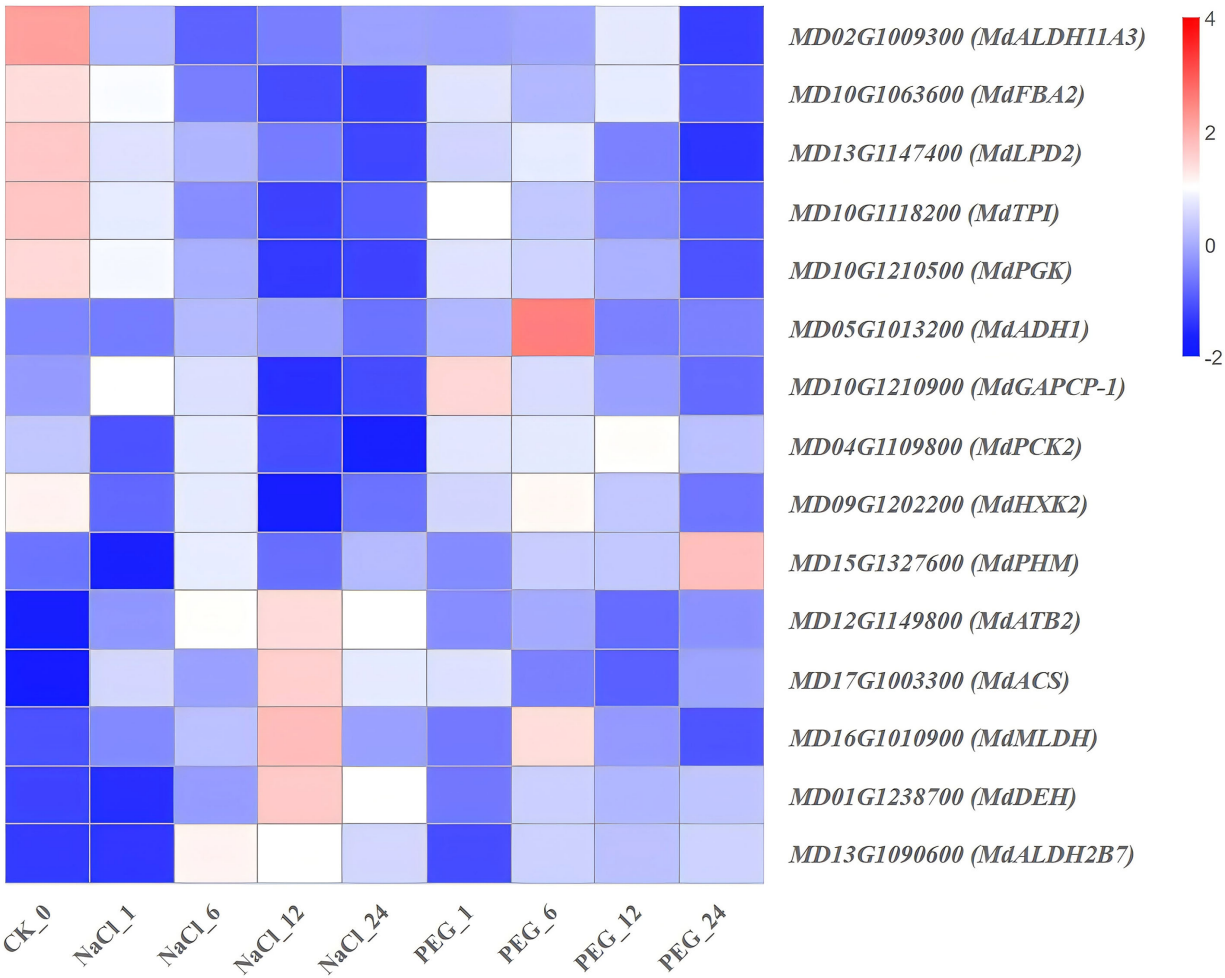
Heat map diagram of the log2 (FPKM) values for genes annotated as glycolysis/gluconeogenesis pathway genes under different treatment groups (control, NaCl, and PEG).

**Figure 11:**
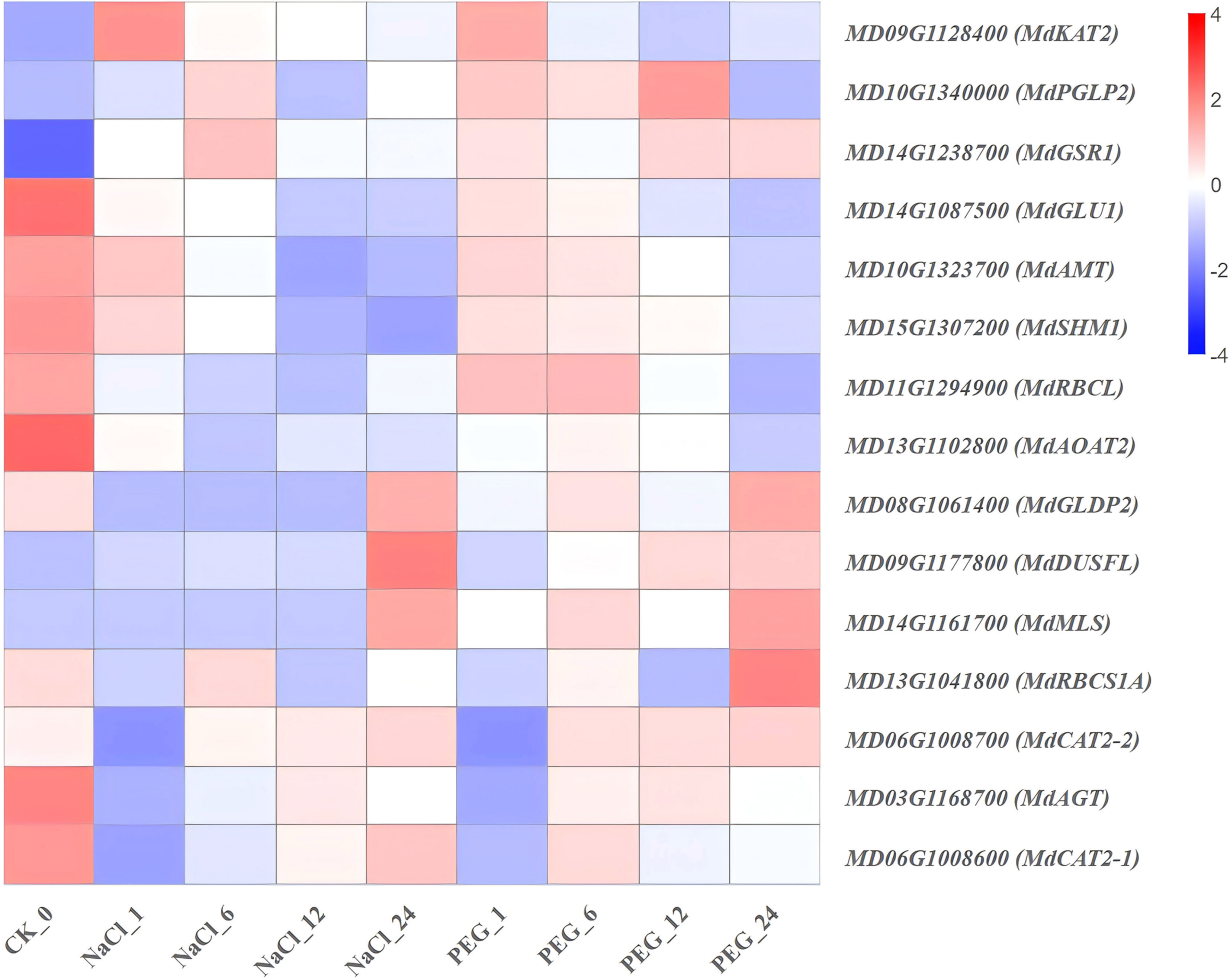
Heat map diagram of the log2 (FPKM) values for genes annotated as glyoxylate and dicarboxylate metabolism-pathway genes under different treatment groups (control, NaCl, and PEG).

**Figure 12:**
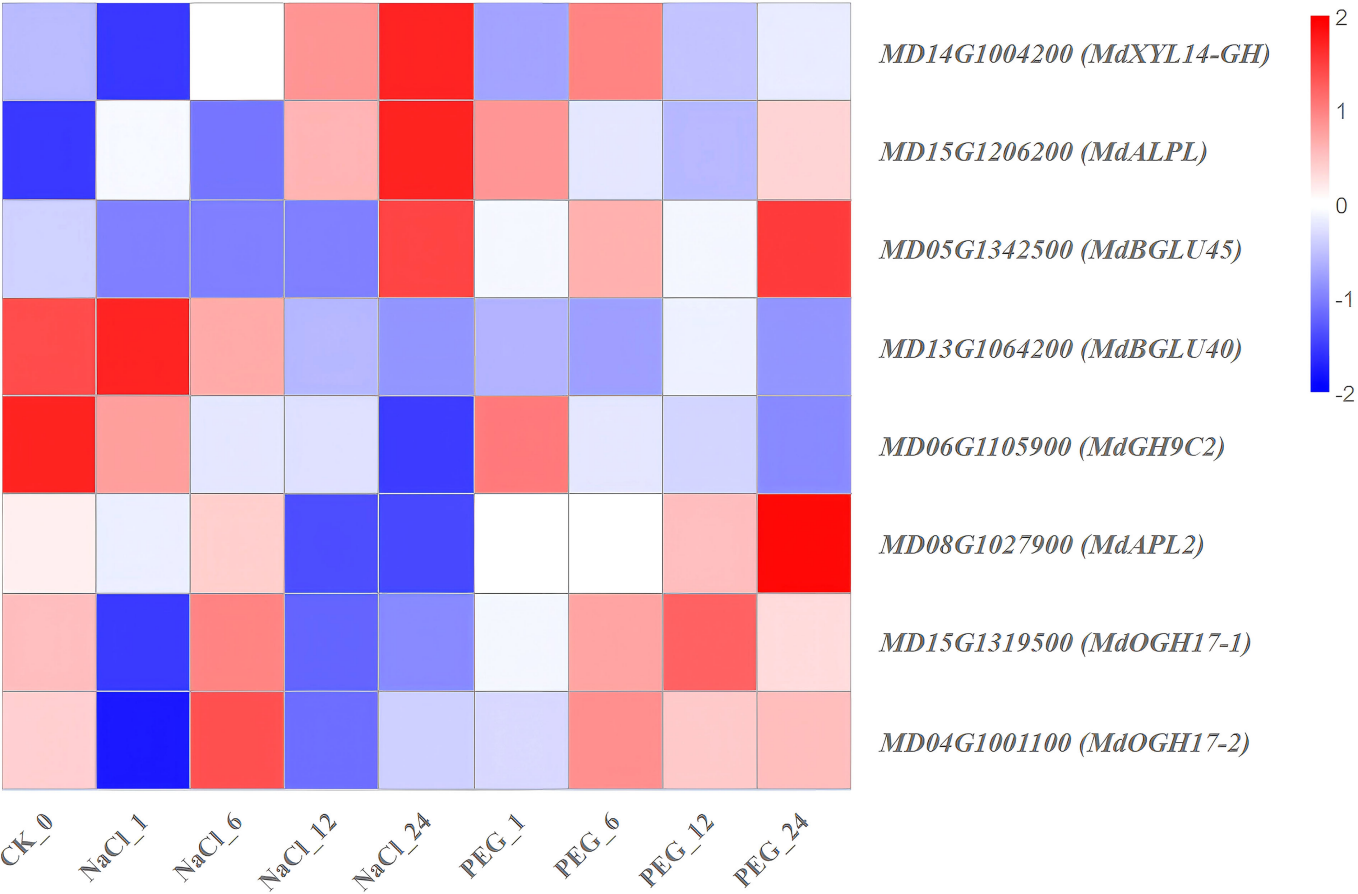
Heat map diagram of the log2 (FPKM) values for genes annotated as starch and sucrose metabolism-pathway genes under different treatment groups (control, NaCl, and PEG).

**Figure 13:**
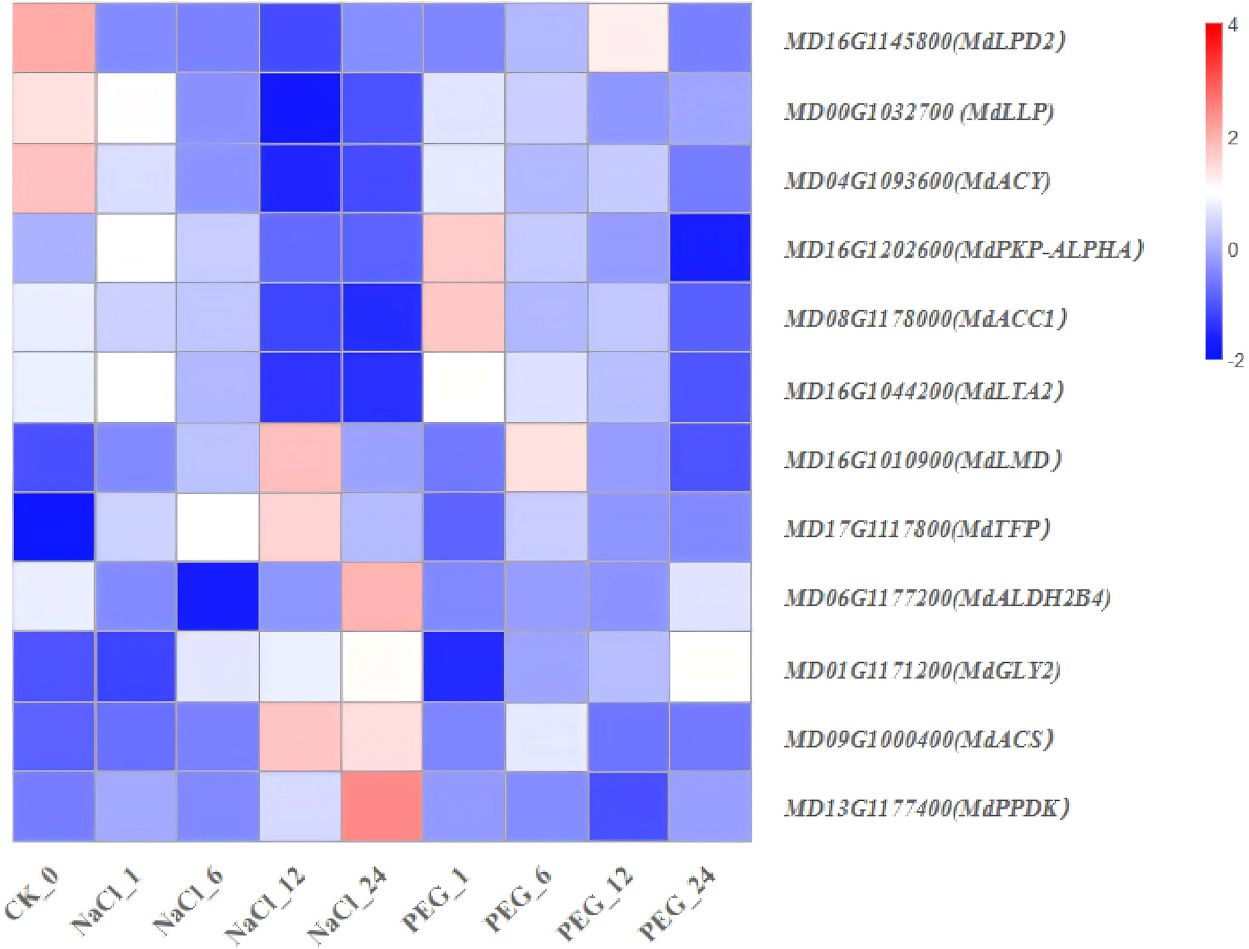
Heat map diagram of the log2 (FPKM) values for genes annotated as pyruvate metabolism-pathway genes under different treatment groups (control, NaCl, and PEG).

**Figure 14:**
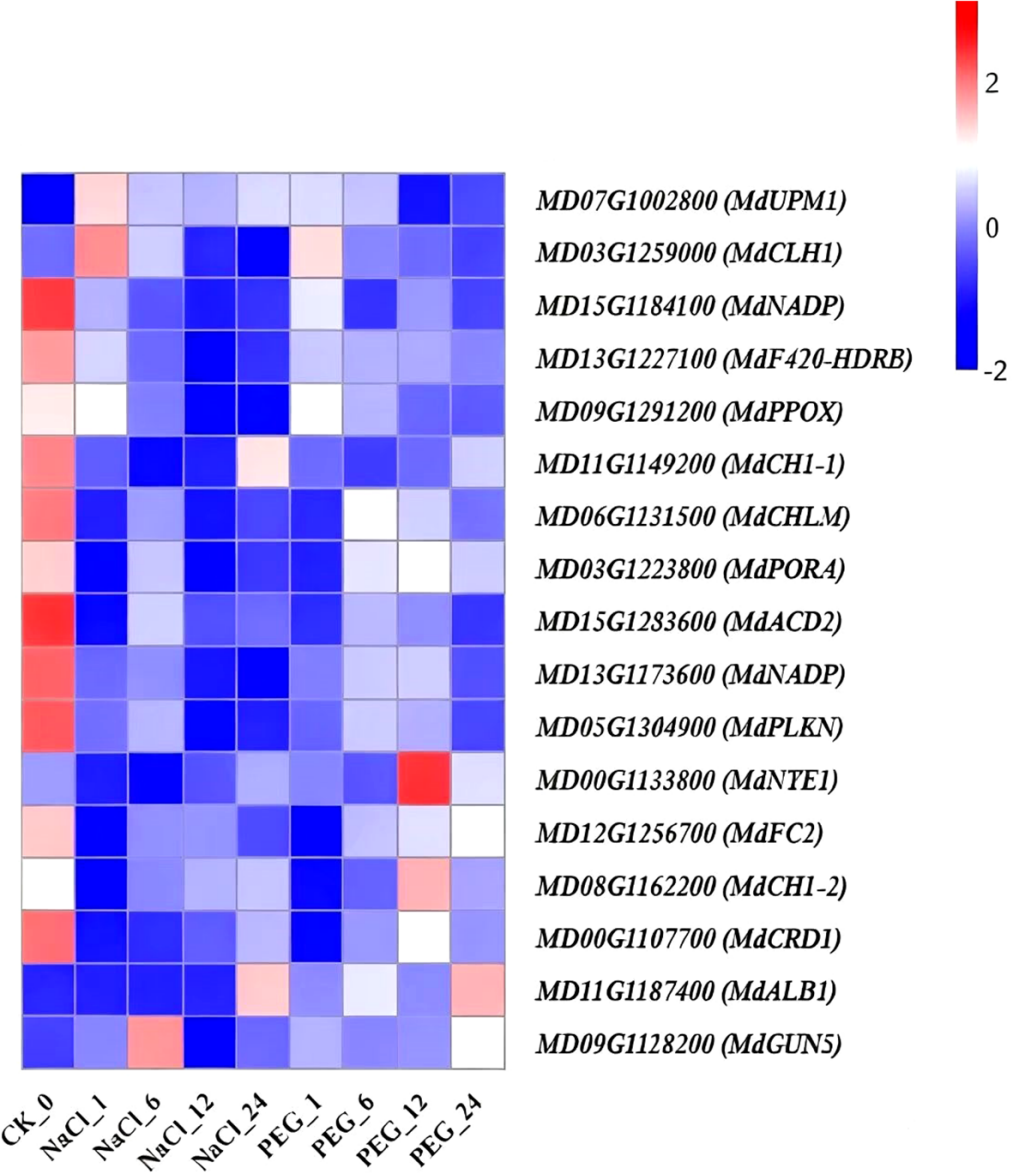
Heat map diagram of the log2 (FPKM) values for genes annotated as porphyrin and chlorophyll metabolism-pathway genes under different treatment groups (control, NaCl, and PEG).

**Figure 15:**
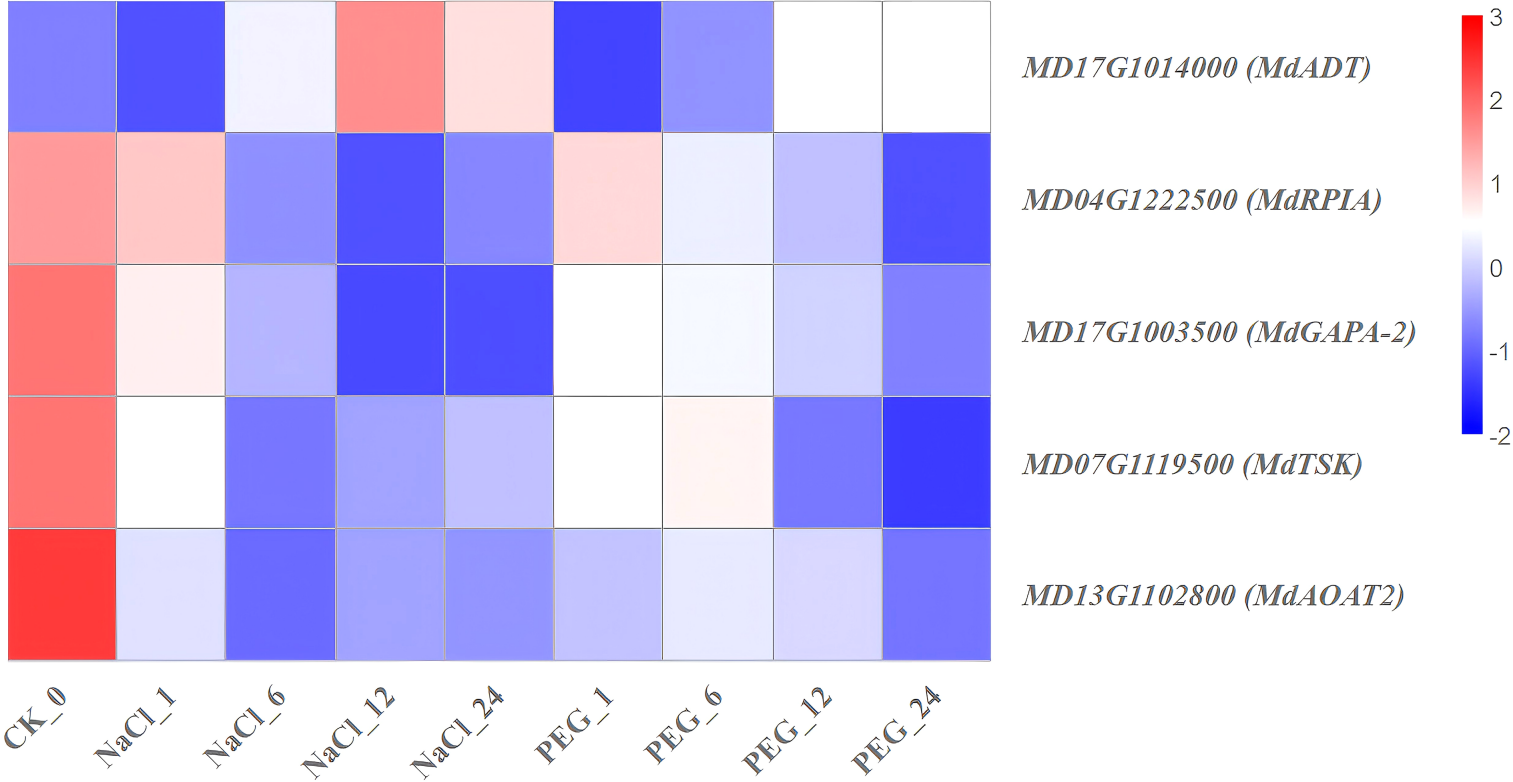
Heat map diagram of the log2 (FPKM) values for genes annotated as carbon fixation genes in photosynthetic organisms under different treatment groups (control, NaCl, and PEG).

**Figure 16:**
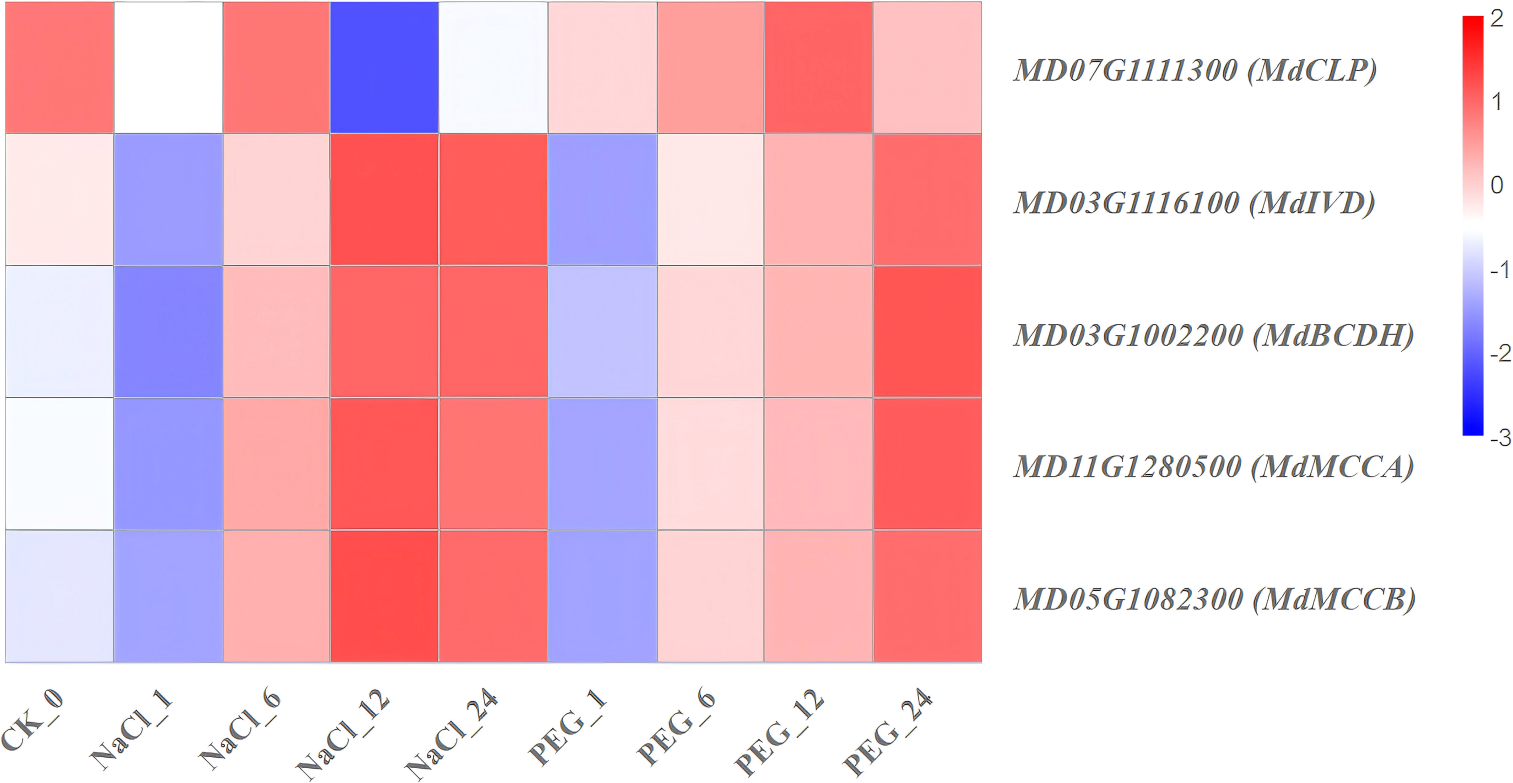
Heat map diagram of the log2 (FPKM) values for genes annotated as valine, leucine, and isoleucine degradation-pathway genes under different treatment groups (control, NaCl, and PEG).

Significant enrichment of channel mainly includes plant hormone signal transduction, photosynthesis, amino sugar and nucleotide sugar metabolism, glycolysis/Gluconeogenesis, glyoxylate and dicarboxylate metabolism, starch and sucrose metabolism, pyruvate metaolism, porphyrin and chlorophyll metabolism, carbon fixation in photosynthetic organisms, valine, leucine and isoleucine degradation-related genes were selected from the heavily enriched KEGG pathways (**Figure 6C**). Finally, the selected DEGs from pathways were divided into ten categories, and the expression profiles of the representative genes in each group were analyzed in **Figures 7**–**16**.

### Expression characteristics of genes involved in the plant hormone signal-pathway

Plant hormones play a crucial role in regulating physiological processes, and auxin are key hormones governing root development, this study investigated the effects of NaCl (salt) and PEG (simulated drought) treatments on apple, and further analyzed the expression profiles of plant hormone signal transduction-related genes (**Supplementary Table 5**; **Figure 7**). The dynamic expression profiles (FPKM values) of 18 hormone signaling-related genes in under NaCl (salt) and PEG (simulated drought) stresses. These genes were categorized into 4 groups based on their temporal expression trends.

(1) NaCl-PEG co-upregulated type (sustained upregulation): *MdSNF1*, *MdABRE3*, and *MdRCAR1* maintained 2-3-fold higher expression levels at 6–24 h under both NaCl and PEG treatments (**Figure 7**). This suggests that the core *ABA-SnRK2-AREB* axis was synergistically activated, serving as a key driving module for root-stock cross-tolerance (**Figure 7**). (2) NaCl-specific burst type (early peak): *MdJAZ1* (JA signal repressor) surged to 10-fold at 1 h under NaCl treatment, followed by a subsequent decline (**Figure 7**); *MdNPR3–1* also exhibited a ‘single-peak’ pattern (**Figure 7**). (3) Post-PEG recovery type (first down-regulated then up-regulated): *MdPIL6*, *MdPP2CA* and *MdCOI1* showed significant recovery at 12–24 h under PEG treatment (1.5-2-fold of CK) (**Figure 7**), while their expression remained persistently low under NaCl treatment. This reflects that drought maintains long-term responses through an ‘ABA de-repression-reactivation’ loop, which is an important marker for apple drought tolerance recovery. (4).Background homeostasis type: Genes including *MdCYCD3;1*, *MdXTR6*, *MdAHP4*, *MdDFL2*, *MdSAUR*, *MdBAK1*, *MdCAP*, *MdTGA6*, *MdETT* and *MdNPR3–2* exhibited expression fluctuations<2-fold. These genes were regarded as the hormone signal background maintenance module and did not participate in salt/drought-specific regulation (**Figure 7**).

In conclusion, *MdSNF1* and *MdAREB3* can serve as core markers for salt-drought common tolerance in s; *MdJAZ1* is an early diagnostic gene for salt tolerance; and the post-drought recovery of *MdPIL6* and *MdPP2CA*, as a key indicator for evaluating drought tolerance potential of apple.

### Expression characteristics of genes involved in photosynthesis pathway

High-depth transcriptome sequencing (FPKM) was used to resolve the dynamic expression patterns of 23 photosynthesis pathway-related genes in apple under NaCl (salt) and PEG (simulated drought) treatments. The results were categorized into 3 classes (**Supplementary Table 6**; **Figure 8**):

(1) NaCl-PEG dual inhibition type (12 genes): This group primarily comprises core components of the photosynthetic electron transport chain, including *MdPETE1* (plastocyanin), *Md2Fe-2S*, *MdPSAD-2*, *MdPSAG*, *MdPSAH2*, *MdPSAL, MdPSB27, MdPETC* and *MdPSBO2* as well as three ATP synthase subunits (*MdATPC1*, *MdATPD*, *MdATPF*) (**Figure 8**). Compared with the control (0 h), these genes exhibited a sharp 50.0-70.0% down-regulation as early as 1 h after NaCl treatment (**Figure 8**). A similar pattern of decreased expression was observed under PEG treatment, with the magnitude and temporal dynamics of down-regulation consistent with the NaCl stress response (**Figure 8**), indicating a conserved inhibitory effect of both stresses on these photosynthetic machinery-related genes. (2) Salt stress-specific down-regulated genes (3 genes): *MdPSAN*, *MdPSBR* and the light-harvesting protein-encoding gene *MdLIL3:1* showed a decreasing trend under NaCl stress, with ≥80.0% downregulation at 24 h vs. 0 h (**Figure 8**). In contrast, these genes only exhibited a 30.0-40.0% downregulation during the same period under PEG treatment (**Figure 8**). (3) Genes with “PEG-specific down-up, NaCl-persistent down” pattern (3 genes): *MdPSBP-1*, *MdPSBY*, and *MdPSBP-1* showed decreased expression from 0 to 7 h, followed by a significant rebound (>1.5-fold) from 12 to 24 h under PEG treatment (**Figure 8**). In contrast, their expression was continuously suppressed under NaCl treatment (**Figure 8**). (4) Additionally, the other genes were no significant temporal regularity (**Figure 8**; **Supplementary Table 6**).

Taken together, electron transport chain genes (*MdPETE1*, *Md2Fe-2S*, *MdPSAD-2*, *MdPSAG*, *MdPSAH2*, *MdPSAL*, *MdPSB27*, *MdPETC and MdPSBO2*) and ATP synthase subunits (*MdATPC1*, *MdATPD*, *MdATPF*) were synchronously inhibited by≥50.0% under both NaCl and PEG treatments, making them potential functional target genes for apple stress tolerance research.

### Expression characteristics of genes involved in amino sugar and nucleotide sugar metabolism-pathway

To characterize the response of apple in amino sugar and nucleotide sugar metabolism, 21 related genes were analyzed via high-depth transcriptome sequencing (FPKM). These genes exhibited four distinct expression patterns under NaCl (salt stress) and PEG (simulated drought stress) treatments (**Figure 9**; **Supplementary Table 7**).

(1) NaCl-PEG dual-repressed type (*MdSIS*, *MdGME*, *MdGlcNAc1pUT1*, *MdGalAK*, *MdXYL4*, *MdRHM1*, *MdUGE5*) showed continuous down-regulation under both treatments (**Figure 9**). These genes mainly encode enzymes for nucleotide sugar synthesis and modification. (2) NaCl-PEG dual-induced type (*MdPGM*) was significantly up-regulated at all time points under NaCl and PEG treatments (**Figure 9**). (3) NaCl-PEG non-responsive type (*MdNRS/ER*, *MdNADP1*, *MdUDP6*, *MdAPL2*, *MdUXS6*, *MdUXS6-1*, *MdAXS2*, *MdASD1*, *MdNDS*, *MdAGPP*, *MdGAE1*, *MdNADP3*) exhibited no significant temporal regularity in expression—falling between the dual-repressed and drought-induced types (**Figure 9**).

In conclusion, under NaCl and PEG treatments, apple adopt a three-tiered regulatory strategy (“Down-regulate growth-related synthesis, up-regulate cell wall remodeling, and differentiate regulate redox homeostasis”) via the aforementioned genes to coordinately cope with both stresses. Among these, *MdSIS*, *MdGME*, *MdGlcNAc1pUT1*, *MdGalAK*, *MdXYL4*, *MdRHM1*, and *MdUGE5* are promising common candidate targets for improving salt and PEG resistance in apple breeding.

### Expression characteristics of genes involved in glycolysis/gluconeogenesis pathway

To identify key functional genes for molecular breeding, high-throughput transcriptomics was used to analyze the dynamic response patterns of glycolysis/gluconeogenesis pathway genes in apple; these patterns are shown in **Supplemetary Table 8** and **Figure 10**. Furthermore, 15 related genes exhibited three distinct expression patterns under NaCl (salt) and PEG (simulated drought) stresses:

(1) NaCl-specific burst type (early sharp induction): *MdDEH*, *MdATB2*, and *MdALDH2B7* exhibited a drastic 5-50-fold up-regulation at 6–12 h of NaCl treatment (**Figure 10**), indicating that salt ion burst activates the NAD(P)-dependent glyoxylate detoxification modul-a key response for s to transiently mitigate salt-induced oxidative stress. (2) PEG-specific accumulation type (sustained up-regulation): *MdADH1* and *MdMLDH* maintained high expression (5-20-fold) post PEG (6 h) (**Figure 10**), demonstrating that drought promotes sustained activation of the “pyruvate-lactate” fermentation branch to enhance hypoxic tolerance and maintain energy homeostasis in *apple*. (3) Dual-stress repression type (sustained downregulation): Core glycolytic enzymes (*MdFBA2*, *MdPGK*, *MdTPI*) showed a “stepwise” Down-regulation under both NaCl and PEG treatments (≥60.0% reduction at 24 h (**Figure 10**), suggesting that salt and drought jointly inhibit carbon flux through the main glycolytic pathway, redirecting carbon skeletons to osmoprotection and detoxification bypasses. (4) The remaining 7 genes (*MdALDHI1A3*, *MdPCK2*, *MdHXK2*, *MdGAPCP-1*, *MdLPD2*, *MdPHM*, *MdACS*) showed no significant temporal regularity in expression under both treatments (**Figure 10**). These genes were considered a background maintenance module and not involved in salt/drought-specific regulation of apple.

In conclusion, *MdDEH* and *MdALDH2B7* are potential early diagnostic markers for salt tolerance; *MdADH1* and *MdALDH2B7* are indicators of drought tolerance potential; and coordinated downregulation of central glycolytic enzymes (*MdFBA2*, *MdPGK*, *MdTPI*) are a core event in carbon metabolism reprogramming of apple under combined salt and drought stresses.

### Expression characteristics of genes involved in glyoxylate and dicarboxylate metabolism-pathway

To dissect the response of apple at the glyoxylate and dicarboxylate metabolism level, high-throughput transcriptome data (FPKM) were used to perform stress time-series clustering on 15 core genes of this pathway (**Supplementary Table 9**; **Figure 11**). The results are as follows:

(1) Dual-treatment sustained repression type genes (*MdAGT*, *MdCAT2-1*, *MdAMT*, *MdRBCL*, *MdAOAT2*, *MdGLU1*, *MdSHM1*) exhibited significant downregulation under both NaCl and PEG treatments, with 24 h expression at 8.0-35.0% of the 0 h level (**Supplementary Table 9**; **Figure 11**). Functionally, they encode key photorespiratory enzymes (serine hydroxymethyltransferase, glycine decarboxylase P-protein) and rubisco large/small subunits. (2) NaCl-PEG dual-induced, early-peaking type genes (*MdCAT2–2* and *MdKAT2*) were significantly up-regulated under NaCl and PEG treatments, with expression peaking at 6 h (max. induction of 2.1-2.7-fold vs. 0 h) (**Figure 11**). (3) NaCl-PEG non-patterned type genes (*MdGLDP2*, *MdDUSFL*, *MdPGLP2*, *MdRBCS1A*, *MdMLS*, *MdGSR1*) were observed no significant temporal regularity in their expression levels (**Figure 11**).

In conclusion, apple coordinately regulate the glyoxylate-dicarboxylate metabolism network via a dual adaptive strategy: “inhibit photorespiration while enhancing ROS scavenging and acetyl-CoA supply”. Consequently, *MdAGT*, *MdCAT2-1*, *MdAMT*, *MdRBCL*, *MdAOAT2*, *MdGLU1*, *MdSHM1*, *MdCAT2-2*, and *MdKAT2* are potential common candidate targets for improving salt and drought tolerance in apple molecular breeding.

### Expression characteristics of genes involved in starch and sucrose metabolism-pathway

To dissect salt and drought tolerance mechanisms of apple from the perspective of carbon source reallocation, transcriptome time-series analysis was conducted on 8 starch-sucrose metabolism-related genes (**Supplementary Table 10**; **Figure 12**). These genes exhibited 3 distinct expression patterns, with the following results:

(1) Salt-drought co-repressed type (sustained down-regulation): *MdGH9C2* and *MdBGLU40* showed a “stepwise” decrease under both stresses (≥80.0% down-regulation at 24 h vs. 0 h) (**Figure 12**). (2) Salt-specific burst type (early sharp upregulation): *MdAPL2* (large subunit of ADPG pyrophosphorylase) exhibited a sharp 2.2-fold up-regulation at 12–24 h under NaCl treatment, accompanied by synchronous up-regulation of *MdALPL* and *MdOGH17-1* (**Figure 12**). (3) Drought-specific recovery type (initial downregulation followed by upregulation): *MdOGH17–2* was repressed at 1 h under PEG treatment, followed by significant recovery at 12–24 h (1.3-fold vs. CK), while its expression remained persistently low under NaCl treatment (**Figure 12**). This indicates that drought accelerates sucrose redistribution by reactivating O-glycoside hydrolase, thereby supporting drought tolerance recovery in *apple*. (4) *MdBGLU45* and *MdXYL14-GH* showed FPKM fluctuations <2-fold with no significant regularity, and were considered the background maintenance module (**Figure 12**).

In conclusion, coordinated downregulation of *MdGH9C2* and *MdBGLU40* are a common marker of salt-drought co-inhibition of carbon flux; *MdAPL2* is a potential early diagnostic gene for salt tolerance; and post-drought recovery of *MdOGH17–2* is a key indicator of drought tolerance potential in *apple*.

### Expression characteristics of genes involved in pyruvate metabolism-pathway

To dissect the pyruvate metabolism-related response of apple, time-series stress analysis was conducted on 12 core pathway genes via high-throughput transcriptome data (FPKM) (**Supplementary Table 11**; **Figure 13**). Results are as follows:

(1) “NaCl-PEG dual-upregulated type” (*MdACS*, *MdLMD*, *MdTFP*): These genes showed significant time-dependent upregulation under both treatments, with their expression at 1–24 h up-regulated relative to 0 h (**Figure 13**). (2) “NaCl-PEG dual-downregulated type” (*MdLLP*, *MdGLY2*, *MdACY*, *MdLPD2*): These genes were significantly down-regulated under both NaCl and PEG treatments, with their 24 h expression reduced to 5.0%-60.0% of the 0 h level (**Figure 13**). (3) “Salt-drought non-significant change type” (*MdALDH2B4*, *MdACC1*, *MdPPDK*, *MdLTA2*, *MdPKP-ALP*): No significant temporal regularity were observed in their expression (**Figure 13**).

Based on gene expression patterns and functions, this study preliminarily demonstrates that apple reprogram carbon flux and conserve energy by activating acetyl-CoA synthesis/ROS scavenging and inhibiting ineffective pyruvate consumption. The dual-up-regulated *(MdACS*, *MdLMD*, *MdTFP*) and dual-down-regulated genes (*MdLLP*, *MdGLY2*, *MdACY*, *MdLPD2*) can serve as common molecular markers for salt and drought tolerance, providing candidates for subsequent genetic improvement.

### The expression of porphyrin and chlorophyll metabolism pathway-related genes in the RNA-seq data

Time-series transcriptome analysis was performed on 17 porphyrin/chlorophyll metabolism genes in apple under NaCl and PEG treatments (0 h as control, CK), with their expression changes compared over 0–24 h (**Supplementary Table 12**; **Figure 14**). These genes were categorized into 3 groups based on their expression trends:

“NaCl-PEG dual-downregulated type” (*MdACD2*, *MdNADP-1/MdNAD-2*, *MdF420-HDRB*, *MdFC2*, *MdCH1-1*, *MdPPOX*, *MdCHLM*, *MdPLKN*, *MdPORA*, *MdCRD1*): These genes showed sustained downregulation under both treatments, with 24 h expression reduced to 20.0-50.0% of CK. Among them, *MdPLKN* had the mildest down-regulation (1.8-fold) under PEG, while *MdNADP-2* showed the most significant downregulation (4.5-fold) under NaCl (**Figure 14**). (2) “NaCl-PEG dual-upregulated, NaCl-stable type”: *MdUPM1* was significantly upregulated at 1–24 h vs. CK under both NaCl and PEG treatments (**Figure 14**). (3) “NaCl-PEG non-significant change type” *(MdALB1*, *MdCUN5*, *MdCH1-2*, *MdCLH1*, *MdNYE1*): These genes showed <1.8-fold expression variation vs. CK under both treatments, with no significant temporal regularity (**Figure 14**).

In conclusion, apple maintain chlorophyll homeostasis under salt/drought via two strategies: down-regulating dual-down-regulated genes (e.g., *MdACD2*, *MdNADP-1/MdNAD-2*, *MdF420-HDRB*, *MdFC2*, *MdCH1-1*, *MdPPOX*, *MdCHLM*, *MdPLKN*, *MdPORA*, *MdCRD1*) to reduce precursor consumption and photo-oxidative damage, and upregulating *MdUPM1* (salt/drought-induced) to sustain synthesis. *MdUPM1* is a priority candidate for drought breeding; dual-down-regulated genes serve as common salt/drought targets to mediate chlorophyll metabolism and enhance resistance.

### Expression characteristics of genes involved in photosynthetic organisms carbon fixation-pathway

In this study, transcriptome analysis of apple under NaCl and PEG treatments identified 5 genes involved in the photosynthetic carbon fixation pathway, which were categorized into 3 groups based on their expression trends (**Supplementary Table 13**; **Figure 15**):

(1) “NaCl-PEG dual-repressed type” genes (*MdRPIA*, *MdTSK*, *MdAOAT2*, *MdGAPA-2*) were significantly down-regulated under both NaCl and PEG treatments compared with the 0 h (**Figure 15**). Specifically, *MdRPIA* exhibited a 3.9-fold down-regulation under NaCl treatment and a 5.5-fold down-regulation under PEG treatment, while *MdGAPA-2* showed a 4.6-fold down-regulation under NaCl treatment and a 3.5-fold down-regulation under PEG treatment (**Figure 15**). (2) For the “NaCl-PEG non-significant change type”, *MdADT* showed no significant temporal expression pattern, and its expression profile fell between that of the dual-repressed and drought-induced types (**Figure 15**).

In conclusion, *MdRPIA*, *MdTSK*, *MdAOAT2*, and *MdGAPA-2* are common repressed targets in the photosynthetic carbon fixation pathway of apple under salt and drought treatments, and can serve as core candidates for subsequent RNAi/CRISPR-based studies or marker-assisted selection in breeding.

### Expression patterns of genes involved in valine, leucine, and isoleucine degradation-pathway

Branched-chain amino acid (BCAA) degradation is a key hub for energy and carbon skeleton redistribution in apple under NaCl (salt) and PEG (simulated drought) treatments. Transcriptome analysis identified 5 genes in this pathway, grouped into 2 categories by expression trends (**Supplementary Table 14**; **Figure 16**):

(1) Salt-drought co-upregulated type (sustained high expression): *MdMCCA*, *MdMCCB* (methylcrotonyl-CoA carboxylase) and *MdIVD* (isovaleryl-CoA dehydrogenase) maintained 2-4-fold up-regulation at 6–24 h under NaCl and PEG treatments (**Figure 16**). This suggests the BCAA degradation pathway was synergistically activated, providing additional acetyl-CoA and NADH to support energy metabolism and osmotic regulation in apple under treatments. (2) Background fluctuation type: *MdCLP* and *MdBCDH* showed low expression (<25 FPKM) and temporal changes <1.5-fold, with no significant stress response observed (**Figure 16**).

In conclusion, synergistic high expression of *MdMCCA*, *MdMCCB* and *MdIVD* can serve as a functional marker for BCAA degradation activity associated with salt and drought tolerance in *apple.* Their potential to enhance stress tolerance can be verified via enzyme activity assays and transient over-expression experiments in subsequent studies.

## Discussion

### Plant hormone signal transduction: mining stress-responsive core genes in *apple* during NaCl/PEG-treatments

*MdSNF1* and *MdABRE3* showed significant continuous up-regulation under both NaCl and PEG induction (**Figure 7**). Previous studies have shown that *SnRK2.6/open stomata 1* (*OST1*)/*SRK2E*-a member of the Sucrose Non-Fermenting 1 (*SNF1*)-related protein kinase 2 family-plays a pivotal role in abscisic acid (ABA) signaling in *Arabidopsis* guard cells (Liang et al., 2015), and its activity is reported to enhance plant stress tolerance (Punkkinen et al., 2021). Additionally, the abscisic acid-responsive element (ABRE)-a cis-regulatory element-mediates the expression of numerous genes and participates in the responses of higher plants to environmental stresses via ABA during growth (Choi et al., 2000). Collectively, these results suggest *MdSNF1* and *MdABRE3* function as “master switches” in the ABA-dependent pathway in *apple*, synergistically enhancing apple cross-tolerance to salt and drought.

JAZ (Jasmonate ZIM-domain protein)-associated genes showed an expression peak in the early NaCl stress response (**Figure 7**). Functionally, *JAZs* encode key repressors in the jasmonic acid (JA) pathway, are regulated by various abiotic stresses, and play crucial roles in plant stress responses. Furthermore, JAZ proteins inhibit JA-regulated anthocyanin accumulation and trichome initiation (Qi et al., 2011; Chini et al., 2017). The above model indicates that under salt stress, *MdJAZ1* and *MdNPR3–1* preferentially activate the JA-salicylic acid (SA) crosstalk network and initiate cell wall remodeling, whereas this effect is not observed under drought.

In *apple*, *MdPP2CA* specifically responds to PEG treatment, with continuous up-regulation. Previous studies demonstrated that in *Arabidopsis thaliana*, ABA is involved in the dynamic adaptation of root system architecture to environmental stresses, and root growth adaptation is mediated by the ABA receptor-*PP2A* complex (Li et al., 2020); however, the underlying molecular mechanism in *apple* remains unclear. By analyzing *MdPP2CA* expression and combining it with *MdSNF1’s* continuous up-regulation under PEG, we hypothesize that under drought treatment, plants maintain a long-term stress response via an elaborate “ABA-inhibition-reactivation” loop.

### Mining key genes of photosynthesis-associated signaling pathways

As the core module for energy supply and redox homeostasis in fruit tree, transcriptional levels of photosynthesis-related genes directly determine the carbon assimilation capacity and adaptability of apple under NaCl and PEG treatments.

*MdPETE1* (plastocyanin), *Md2Fe-2S*, *MdPSAD-2*, *MdPSAG*, *MdPSAH2*, *MdPSBO2*, *MdPSBR*, and ATP synthase subunits (*MdATPC1*, *MdATPD*, *MdATP*F) responded to both stresses, with sharply down-regulated expression (**Figure 8**). Functionally, these genes participate in photosystem I/II (PSI/II) and the photo-phosphorylation module. In plants, high PSII phosphorylation is essential for regulating photosynthetic membrane macro-organization, modulating membrane protein lateral mobility, and maintaining sustained photosynthetic activity (Fristedt et al., 2009). Thus, transcriptional repression of these genes is likely an early molecular event driving reduced photosynthetic rate in apple and may mediate salt/drought treatments response mechanisms.

*MdPSAN*, *MdPSAL*, *MdPSB27*, *MdPETC*, and light-harvesting protein *MdLIL3:1* showed salt-specific down-regulation (**Figure 8**). In oxygenic photosynthesis, PSI and PSII are critical for light-driven electron transport (Su et al., 2019); PSI is a multisubunit pigment-protein complex mediating electron transfer from plastocyanin to ferredoxin in thylakoid membranes of photosynthetic autotrophs (Bína et al., 2017). These expression patterns suggest salt-induced ion toxicity causes irreversible damage to the PSI peripheral antenna and cell wall-thylakoid junction, making these genes potential salt-specific sensitivity markers.

Additionally, *MdPSBP-1*, *MdPSBY*, and *MdPS* showed initial downregulation followed by up-regulation under PEG treatment, but continuous down-regulation under salt treatment (**Figure 8**). This implies *apple* can rapidly reassemble PSII peripheral proteins to repair drought-induced photoinhibition, whereas this repair mechanism is absent under salt stress.

In summary, electron transport chain genes (*MdPETE1*, *Md2Fe-2S*, *MdPSAD-2*, *MdPSAG*, *MdPSAH2*, *MdPSAL*, *MdPSB27*, *MdPETC*, *MdPSBO2*) and three ATP synthase subunits (*MdATPC1*, *MdATPD*, *MdATPF*) common functional targets regulating drought and salt treatment responses in *apple*.

### Differential genes in amino sugar and nucleotide sugar metabolism drive PEG and NaCl stress adaptation by regulating root development in *apple*

Seven genes (*MdSIS*, *MdGME*, *MdGlcNAc1pUT1*, *MdGalAK*, *MdXYL4*, *MdRHM1*, *MdUGE5*) showed NaCl-PEG dual suppression (**Figure 9**). SIS domain-containing proteins participate in lipopolysaccharide biosynthesis (catalyzing sedoheptulose-7-phosphate to D-glycero-D-manno-heptose-7-phosphate) (Ahmad et al., 2020). In *Arabidopsis*, disrupted N-acetylglucosamine-1-P uridylyltransferase impairs protein N-glycosylation and induces ABA-mediated salt sensitivity (seed germination/early seedling growth) (Chen et al., 2022). L-rhamnose (a plant deoxysugar) exists in cell walls as polymers and modifies specialized metabolites (Jiang et al., 2021). Together with their expression patterns, cell wall precursor supply is transcriptionally suppressed globally in early salt/drought stress-likely linked to stress-induced growth arrest, with the regulatory mechanism to be further explored.

In contrast, *MdPGM* (encoding phosphoglucomutase/phosphomannomutase) was NaCl-PEG dual-induced (**Figure 9**), suggesting *MdPGM* under NaCl and PEG treatments to mediate pectin/hemicellulose side-chain remodeling and mitigate damage. Four genes (*MdAPL2*, *MdAGPP*, *MdUGE5*, *MdASD1*) exhibited “NaCl-suppressed, PEG-stable” expression (**Figure 9**). They mainly regulate starch-nucleotide sugar inter-conversion; reduced ADPGlc-PPase activity inhibits starch synthesis (Ekkehard Neuhaus and Stitt, 1990). Their expression implies that under NaCl stress, carbon sources are prioritized for osmotic regulator synthesis, while under PEG, gene expression is maintained to ensure energy reserves.

Another five genes (*MdNADP1/2/3*, *MdGAE1*, *MdNDS*) exhibited “NaCl-induced, PEG-suppressed” expression patterns (**Figure 9**). All of these genes either contain a Rossmann-fold domain or encode glycosyltransferases, which may potentially enhance salt-induced reactive oxygen species (ROS) scavenging and cell wall reinforcement via *NADPH* supply and sugar donor activation.

In summary, apple adopt a three-tiered strategy (“downregulate growth-related synthesis, up-regulate cell wall remodeling, differentially regulate redox homeostasis”) via these genes to cope with salt/drought stresses. *MdSIS*, *MdGME*, *MdGlcNAc1pUT1*, *MdGalAK*, *MdXYL4*, *MdRHM1*, *MdUGE5*, and *MdPGM* are candidates for future genetic improvement NaCl/PEG resistance in *apple*.

### Glycolysis/gluconeogenesis pathway: transcriptome-screened differential genes mediate drought and salt stress to regulate root development in *apple*

*MdFBA2, MdPGK*, and *MdTPI* exhibited dual suppression under salt and drought stresses (**Supplementary Table 8**; **Figure 10**). Functionally, these genes mediate core steps of glycolysis and encode fructose-bisphosphate aldolase, phosphoglycerate kinase (*PGK*), and triosephosphate isomerase (*TPI*), respectively. Specifically, fructose-bisphosphate aldolase participates in carbohydrate metabolism (Liu et al., 2023); *PGK* is a key glycolytic enzyme that mediates nuclear DNA replication and repair (Kumar et al., 2019); and in *rice*, *OsTPI1.1* a component of *XA3/XA26*-mediated resistance to *Xanthomonas oryzae* pv. *Oryzae*-catalyzes the reversible conversion of dihydroxyacetone phosphate to glyceraldehyde-3-phosphate (Liu et al., 2018).

Combined with their functions and expression patterns, these results indicate apple cope with salt/drought-induced energy crisis by globally suppressing classical glycolytic flux to reduce ATP consumption, achieving a “metabolic brake”.

Based on their expression patterns, *MdFBA2*, *MdPGK*, and *MdTPI* are potential common functional candidates regulating apple responses to salt and drought.

### Transcriptomic genes in glyoxylate-dicarboxylate metabolism mediate apple development under stress

*MdAGT*, *MdCAT2-1*, *MdAMT*, *MdRBCL*, *MdAOAT2*, *MdGLU1*, and *MdSHM1* exhibited “dual-stress sustained suppression” (**Supplementary Table 9**; **Figure 11**). Functionally: *AGT1* (glyoxylate aminotransferase 1) is a peroxisomal aminotransferase central to photorespiration (Liepman et al., 2019); *CAT2* (H_2_O_2_-scavenging catalase) is critical for salt tolerance in *Arabidopsis* (Zhuang et al., 2025); *MdRBCL* encodes RuBisCO, which catalyzes both photosynthetic CO_2_ carboxylation and photorespiratory oxygenation (Andersson et al., 1989); *GLU1* participates in iron deficiency response and long-distance transport in *Arabidopsis* (Cui et al., 2020); mitochondrial *SHMT* (serine hydroxymethyltransferase) associates with glycine decarboxylase to mediate a key photorespiratory CO_2_ cycle step, and *shm1-1* (shm mutant) lacks mitochondrial *SHMT* activity, showing lethal photorespiratory phenotype under ambient CO_2_ in *Arabidopsis* (Voll et al., 2006). Combined with their functions and expression patterns, salt and drought likely coordinate these genes to suppress the photorespiration-glyoxylate cycle, reducing photorespiratory carbon loss and maintaining carbon balance to mitigate stress impacts on apple growth.

In addition, *MdCAT2–2* and *MdKAT2* exhibited dual induction (up-regulation) under NaCl and PEG treatments (**Figure 11**). Most abiotic stresses induce excessive reactive oxygen species (ROS) accumulation, causing oxidative damage to plant cells. Catalase (*CAT*) is critical for plant oxidative stress tolerance by scavenging stress-induced excess H_2_O_2_ (Zhang et al., 2021), and the *CAT* family mediates multiple stress responses in soybean, detoxifying and regulating ROS under various stresses (Muqadas et al., 2022).

Furthermore, thiolases are key lipid metabolism enzymes in prokaryotes and eukaryotes, essential for multiple metabolic pathways (Fang, 2020), though studies in apple remain limited. Combined with their functions and expression patterns, *MdCAT2–2* and *MdKAT2* likely maintain ROS homeostasis and mediate fatty acid β-oxidation—representing a common mechanism for apple s to cope with salt and drought.

### Transcriptomic genes in starch-sucrose metabolism mediate apple development under stress

Under NaCl (salt) and PEG (drought) stresses, *MdGH9C2* and *MdBGLU40* were co-suppressed, with sustained down-regulation during treatments (**Figure 12**). Glycosidases/glycosyl hydrolases (e.g., *GH9C2*) are key members of the carbohydrate-active enzyme (CAZyme) superfamily, catalyzing glycosidic bond hydrolysis in carbohydrates and sugar complexes (Sivaramakrishnan et al., 2024). In plants, family 1 glycoside hydrolase β-glucosidases (BGLU) are pivotal for primary metabolism, critical for secondary metabolism, and essential to activate glycosylation-dependent two-component defense systems (Hannemann et al., 2018). Combining the functions of these genes with their expression in NaCl/PEG-treated apple s, we propose that *MdGH9C2* and *MdBGLU40*—both regulating cell wall polysaccharide hydrolysis-are co-suppressed under stress. This likely represents an adaptive strategy: reducing polysaccharide hydrolysis minimizes carbon loss and maintains osmotic homeostasis, key to alleviating salt/drought damage to apple s.

Under NaCl stress, *MdAPL2* (encoding the large subunit of ADP-glucose pyrophosphorylase), *MdALPL*, and *MdOGH17–1* showed specific synchronous upregulation (**Figure 12**). ADP-glucose pyrophosphorylase (encoded by *APL2*) is a key regulatory enzyme in plant starch synthesis (Ballicora et al., 2004), while O-glycosyl hydrolases (*OGHs*) and organic acid metabolism are linked to drought stress in *maize* (Jin et al., 2019). These results suggest salt-induced ion stress activates the starch synthesis substrate supply module, promoting transient accumulation of osmotically protective polysaccharides in *apple*. Under PEG stress, *MdOGH17–2* expression first decreased then increased (**Figure 12**), indicating drought may accelerate sucrose redistribution by reactivating O-glycosyl hydrolase activity, thereby supporting drought tolerance recovery in *apple*.

In summary, apple achieve carbon flux reallocation and osmotic protection via a dual strategy: “suppressing cellulose degradation and enhancing starch synthesis specifically under salt treatment. Coordinated down-regulation of *MdGH9C2* and *MdBGLU40* is a common marker for carbon flux inhibition under both treatments; *MdAPL2* is a potential early diagnostic gene for salt tolerance; and post-drought recovery of *MdOGH17–2* expression is a key indicator of drought tolerance potential in *apple*.

### Transcriptomic genes in pyruvate metabolism mediate apple development under stress

*MdACS*, *MdLMD*, and *MdTFP* exhibited salt-and-drought dual induction (**Figure 13**). Acetyl-CoA synthetase (*ACS*, encoded by *MdACS*) generates acetyl-CoA (Ac-CoA), though excessive activity consumes ATP and causes plant growth arrest (Zheng et al., 2025). Lactate dehydrogenase (*LMD*, encoded by *MdLMD*) in *soybean* leaves enhances water-logging tolerance via root lactate detoxification and malate/succinate metabolism (Posso et al., 2025). Additionally, thiolases (putatively encoded by *MdTFP*) are key to prokaryotic/eukaryotic lipid metabolism and essential for multiple pathways (Fang, 2020). Integrating their expression patterns and known functions, we propose coordinated enhancement of “carbon-nitrogen-antioxidant” metabolism is a major conserved mechanism for apple to cope with salt/drought.

In contrast, *MdLLP*, *MdGLY2*, *MdACY*, and *MdLPD2* showed salt-and-drought dual suppression (**Figure 13**). These genes act as “brakes” on pyruvate consumption to conserve ATP and carbon skeletons, prioritizing osmotic regulator synthesis and enhancing salt/drought resistance in *apple*.

Collectively, our analysis shows apple s achieve carbon flux reprogramming and energy conservation via a two-tiered strategy: “activate acetyl-CoA synthesis/ROS scavenging while inhibiting futile pyruvate consumption”. The dual-induced (*MdACS*, *MdLMD*, *MdTFP*) and dual-suppressed (*MdLLP*, *MdGLY2*, *MdACY*, *MdLPD2*) genes can serve as molecular markers for apple salt/drought tolerance, providing candidates for subsequent genetic improvement.

### Porphyrin-chlorophyll metabolism genes drive apple stress adaptation

Ten genes (*MdACD2*, *MdNADP-1/MdNAD-2*, *MdF420-HDRB*, *MdFC2*, *MdCH1-1*, *MdPPOX*, *MdCHLM*, *MdPLKN*, *MdPORA*, *MdCRD1*) exhibited a “salt-drought dual-repression” pattern. Functionally: *ACCELERATED CELL DEATH6* (*ACD6*) is a key broad-spectrum resistance component (Zhang et al., 2019); NAD(P)-binding rossmann-fold superfamily proteins mediate chitosan-induced peroxidase activity and lipoxygenase expression in Physcomitrella patens (Marttinen et al., 2023); Pheophorbide a oxygenase (PAO) family proteins (with a rieske [2Fe-2S] domain) degrade chlorophyll into species-specific linear tetrapyrroles via the PAO/chlorophyll breakdown pathway (Das et al., 2018). These genes collectively mediate cell death regulation, coenzyme/magnesium-porphyrin methylation, and P-loop ATPase functions. Their coordinated repression likely reduces chlorophyll precursor consumption and alleviates photooxidative damage, thereby enhancing salt/drought resistance in apple tissues.

By contrast, *MdUPM1* exhibited a “NaCl-PEG dual-induction” pattern. Functionally, it mediates uroporphyrin methylation and protochlorophyllide reduction; its activation maintains chlorophyll synthesis flux, compensating for water loss-induced photosynthetic attenuation to enhance apple drought resistance.

This study applied NaCl and PEG treatments to identify shared stress-responsive genes. Collectively, apple maintain chlorophyll homeostasis under salt/drought via a two-tiered strategy: (1) Down-regulate “dual-repression genes” to reduce chlorophyll precursor consumption and photooxidative damage; (2) Up-regulate the “dual-induction gene”-*MdUPM1* to sustain chlorophyll biosynthesis. These genes thus serve as shared targets mediating chlorophyll synthesis/degradation, enhancing salt/drought resistance in *apple*.

### Carbon fixation (photosynthetic organisms): transcriptomic genes mediate stress regulation of apple development

*MdRPIA*, *MdTSK*, *MdAOAT2*, and *MdGAPA-2* exhibited a “NaCl-PEG dual-repression” pattern. Functionally: Ribose 5-phosphate isomerase A (*RPIA*) catalyzes ribose 5-phosphate hydrolysis and participates in CO_2_ fixation (Ju et al., 2020); transketolase (*TSK*) is a key pentose phosphate pathway enzyme (Georges et al., 2024); cis-acting elements are essential for light regulation of nuclear genes encoding chloroplastic glyceraldehyde 3-phosphate dehydrogenase A subunit (*GAPA*) in *Arabidopsis* (Park et al., 1996). These genes respectively catalyze ribulose-5-phosphate isomerization, glyceraldehyde-3-phosphate dehydrogenation, and transketolation. Their synchronous down-regulation reduces invalid carbon skeleton leakage and prioritizes osmotic regulator synthesis, making them shared “carbon-saving” targets for apple to cope with salt/drought stress.

This study applied NaCl and PEG treatments during apple rooting to identify such shared functional genes. In conclusion, *MdRPIA*, *MdTSK*, *MdAOAT2*, and *MdGAPA-2* are shared repressive targets in the photosynthetic carbon fixation pathway of apple under NaCl/PEG treatments. They serve as core candidates for subsequent RNAi/CRISPR-based studies or molecular marker-assisted breeding.

### VLI degradation genes drive apple stress adaptation

This study applied NaCl and PEG treatments during apple rooting to identify shared stress-responsive genes. Among them, *MdMCCA*, *MdMCCB*, and *MdIVD* exhibited a “salt-drought dual-induction” pattern, emerging as shared target genes under these stresses (**Figure 16**).

Functionally: 3-methylcrotonyl-CoA carboxylase (*MCC*) is a biotin-dependent mitochondrial enzyme essential for leucine catabolism in most organisms (Hu et al., 2023); in *Arabidopsis*, the biotinylated subunit encoded by *AtMCCA* enhances carbonate stress resistance (Wang et al., 2022); putative isovaleryl-CoA dehydrogenase (encoded by IVD) localizes to mitochondria. Notably, *MdMCCA/MdMCCB* encode the α/β subunits of MCC, respectively, while *MdIVD* encodes isovaleryl-CoA dehydrogenase.

Their synchronous upregulation likely accelerates branched-chain amino acid (BCAA) carbon skeleton entry into β-oxidation and the tricarboxylic acid (TCA) cycle-supplying additional ATP and reducing power for plant growth under salt/drought. Thus, *MdMCCA*, *MdMCCB*, and *MdIVD* are shared up-regulated functional genes in the BCAA catabolic pathway of apple, representing core candidates for subsequent molecular breeding and gene editing.

## Conclusion

**Figure 17:**
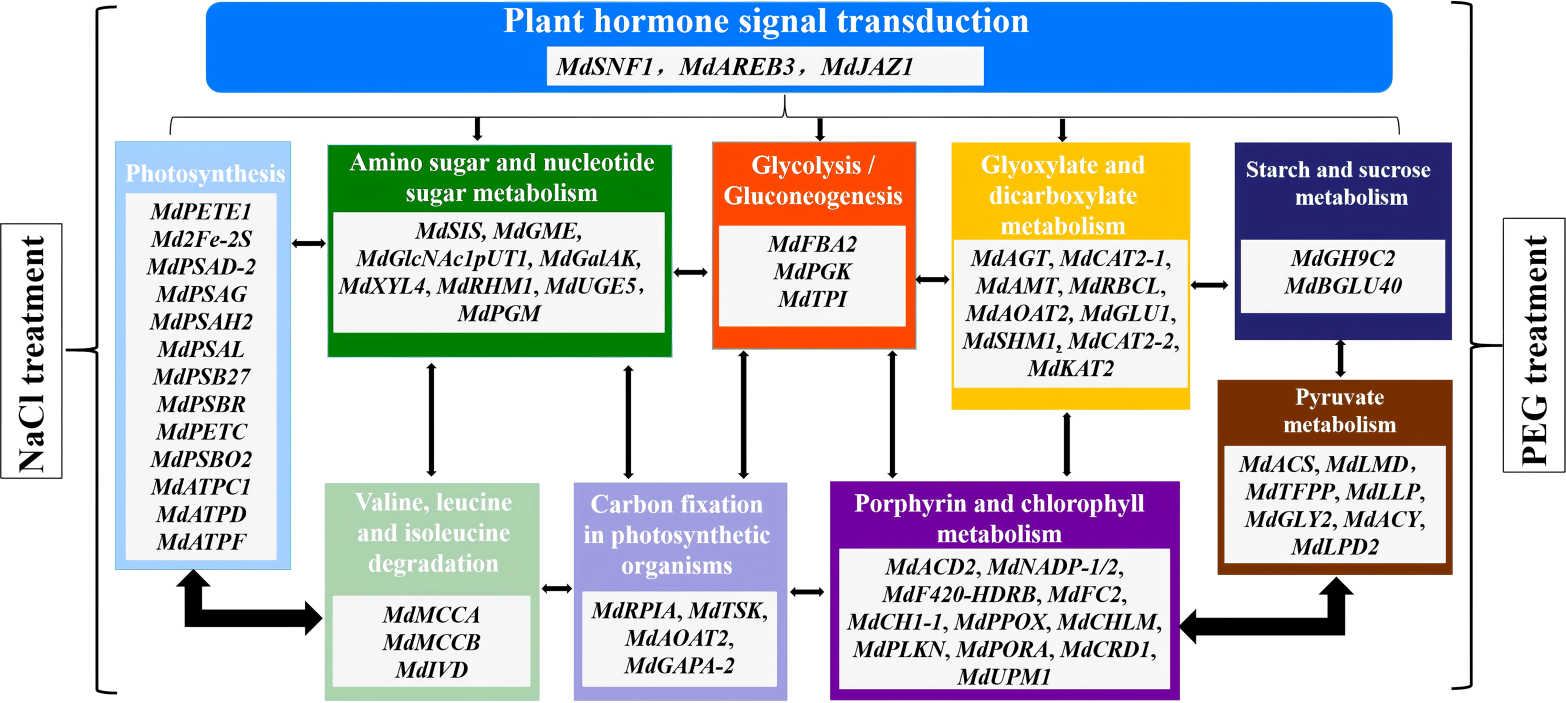
Genes regulatory network of hormone signal transduction & metabolic pathways in apple s under NaCl/PEG treatments. Noet: Modules represent key pathways (e.g., photosynthesis, sugar metabolism) with associated genes; arrows indicate pathway crosstalk. Left/right correspond to NaCl/PEG treatments, respectively.

To clarify the molecular regulatory mechanism of s in coordinately responding to NaCl- and PEG-simulated drought stress, this study constructed a multi-pathway coordinated regulatory network diagram centered on plant hormone signal transduction (**Figure 17**). Based on transcriptome sequencing data (FPKM values) and gene function annotation results. Among them, the core regulatory genes *MdSNF1*, *MdAREB3*, and *MdJAZ1* form an upstream regulatory hub, which regulates 9 key metabolic pathways through cascaded signal transmission. All pathways and their corresponding functional genes are involved in the response process to these two abiotic treatments.

In the metabolic regulation module, the photosynthesis pathway (including 10 genes such as *MdPETE1*, *Md2Fe-2S*, *MdPSAD-2*, *MdPSAG*, *MdPSAH2*, *MdPSAL*, *MdPSB27*, *MdPSBR*, *MdPETC*, *MdPSBO2*, *MdATPC1*, *MdATPD* and *MdATPF*) serves as the core for energy and material supply, providing basic support for stress responses by regulating key steps of light reactions and dark reactions (**Figure 17**). The valine, leucine, and isoleucine degradation pathway (*MdMCCA*, *MdMCCB*, *MdIVD*) generates stress-required intermediates through amino acid metabolic turnover (**Figure 17**). As the core hub of carbon metabolism, the amino sugar and nucleotide sugar metabolism pathway (8 genes including *MdSIS*, *MdGME*, *MdGlcNAclpUT1*, *MdGalAK*, *MdXYL4*, *MdRHM1*, *MdUGE5* and *MdPGM*), glycolysis/gluconeogenesis pathway (*MdFBA2*, *MdPGK* and *MdIPT*), and glyoxylate and dicarboxylate metabolism pathway (9 genes including *MdAGT* and *MdCAT2-1/2-2*) synergistically realize carbon source allocation, energy conversion, and antioxidant synthesis, directly mediating the maintenance of metabolic homeostasis under treatments. In addition, the porphyrin and chlorophyll metabolism pathway (11 genes including *MdACD*2, *MdCHLM* etc) affects light energy utilization efficiency by regulating the synthesis and degradation of photosynthetic pigments; the photosynthetic organism carbon fixation pathway (*MdRPIA*, *MdTSK*, *MdAOAT2* and *MdGAPA-2*) ensures efficient carbon source fixation; while the starch and sucrose metabolism pathway, genes including *MdGH9C2* and *MdBGLU40*) and pyruvate metabolism pathway (7 genes including *MdACS*, *MdLMD*, *MdTFPP*, *MdLLP*, *MdGLY2*, *MdACY* and *MdLPD2*) enhance adaptability to stress through carbohydrate reserve mobilization and key metabolic node regulation, respectively (**Figure 17**).

In conclusion, the key genes of the aforementioned pathways form a tight regulatory network through material exchange, energy transfer, and signal crosstalk. Coordinated by plant hormone signals, they achieve functional synergy to collectively mediate the regulation of growth and development as well as the maintenance of metabolic balance in apple under salt and drought stresses (**Figure 17**). This provides a clear network regulatory framework for deciphering the stress-resistant molecular mechanisms of apple, though the specific functional mechanisms require further in-depth investigation.

## Data Availability

The original contributions presented in the study are publicly available. This data can be found at the National Center for Biotechnology Information (NCBI) using accession number PRJNA908220.

## Supplementary material

Supplementary Figure 1 Sample reproducibility analysis of apple s under CK, NaCl (1/6/12/24 h), and PEG (1/6/12/24 h) treatments. Note: top-Correlation heatmap of samples (color: red→green= correlation coefficient magnitude; values labeled in cells); bottom-Hierarchical clustering plots of replicate samples for each treatment group, showing replicate consistency.

Supplementary Figure 2 Gene set interaction and set size analysis under NaCl **(A)** and PEG **(B)** treatments in apple s. Note: **(A)** Top: Bar chart of interaction sizes for NaCl-related gene sets; Bottom: Set size distribution across NaCl treatment comparison groups (e.g., N1 vs N64, CK vs N1). **(B)** Top: Bar chart of interaction sizes for PEG-related gene sets; Bottom: Set size distribution across PEG treatment comparison groups (e.g., P1 vs P12, CK vs P6).

Supplementary Figure 3 Differential gene expression and set overlap analysis under NaCl/PEG stresses in apple s. **(A, B, D)** Heatmaps showing expression patterns of differential genes (red=upregulation; green=downregulation) across NaCl/PEG treatment groups. **(C)** Venn diagram depicting overlap of upregulated/downregulated gene sets between NaCl and PEG treatments (numbers = gene counts).
