# Supplementary figures and images for: Deciphering intra-connectivity of gene network response to drought and salinity in apple
Source: Front Plant Sci. 2026 Mar 16;17:1763760. doi: 10.3389/fpls.2026.1763760 (PMC13033804; doi:10.3389/fpls.2026.1763760)

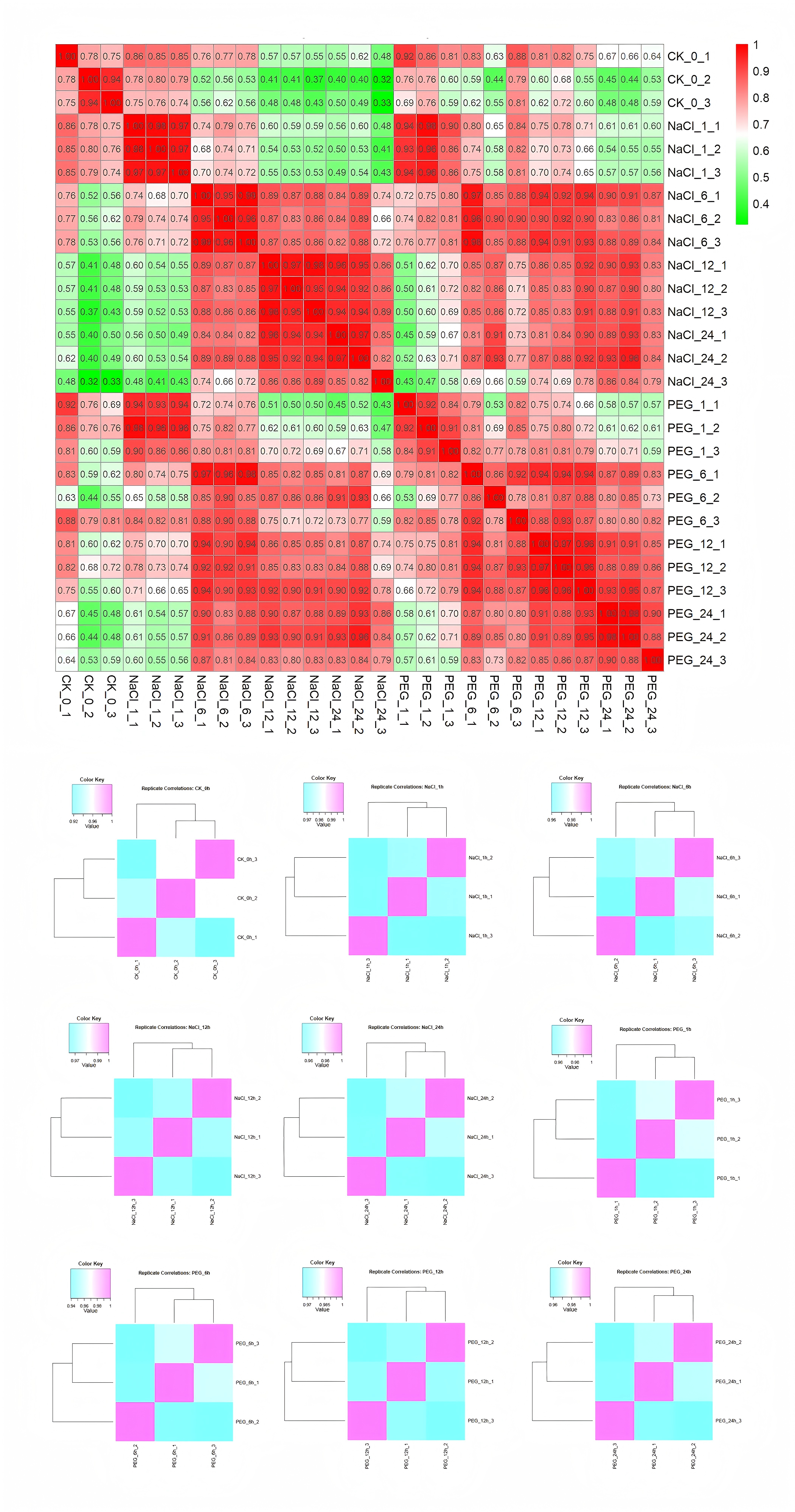

Supplement: Supplementary Figure 1 — Sample reproducibility analysis of apple s under CK, NaCl (1/6/12/24 h), and PEG (1/6/12/24 h) treatments. Note: top-Correlation heatmap of samples (color: red→green= correlation coefficient magnitude; values labeled in cells); bottom-Hierarchical clustering plots of replicate samples for each treatment group, showing replicate consistency. [file Image1.jpeg]

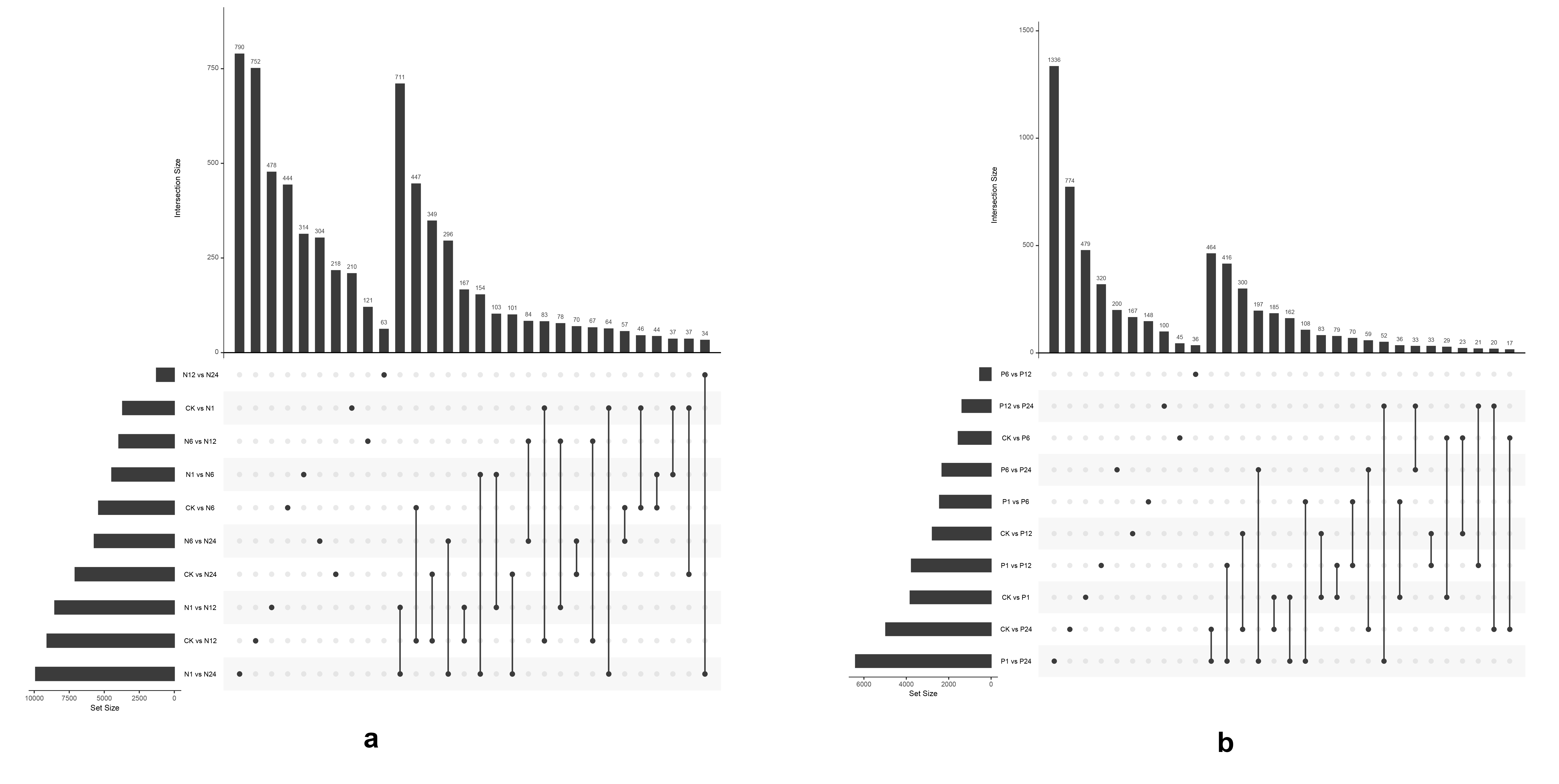

Supplement: Supplementary Figure 2 — Gene set interaction and set size analysis under NaCl (A) and PEG (B) treatments in apple s. Note: (A) Top: Bar chart of interaction sizes for NaCl-related gene sets; Bottom: Set size distribution across NaCl treatment comparison groups (e.g., N1 vs N64, CK vs N1). (B) Top: Bar chart of interaction sizes for PEG-related gene sets; Bottom: Set size distribution across PEG treatment comparison groups (e.g., P1 vs P12, CK vs P6). [file Image2.jpeg]

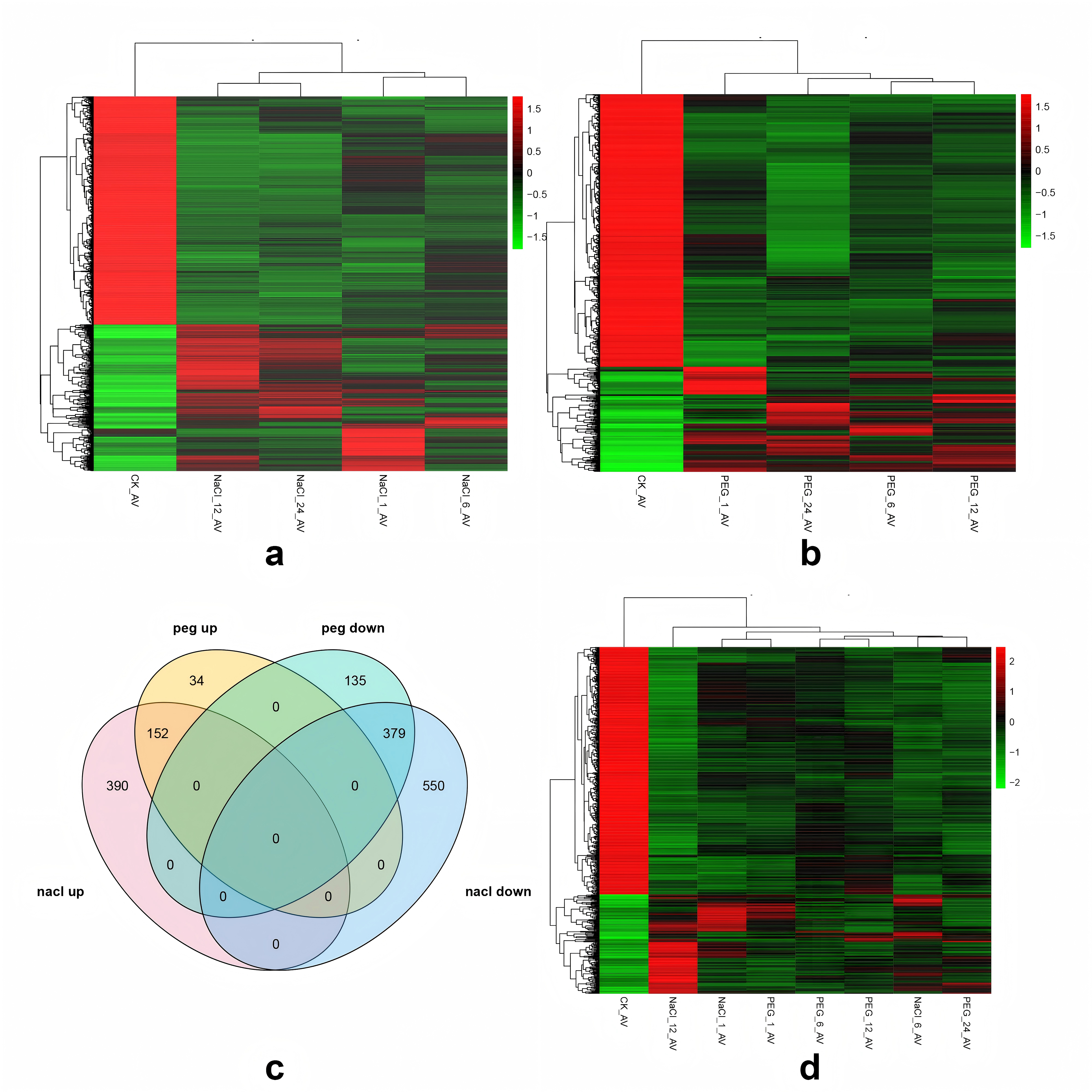

Supplement: Supplementary Figure 3 — Differential gene expression and set overlap analysis under NaCl/PEG stresses in apple s. (A, B, D) Heatmaps showing expression patterns of differential genes (red=upregulation; green=downregulation) across NaCl/PEG treatment groups. (C) Venn diagram depicting overlap of upregulated/downregulated gene sets between NaCl and PEG treatments (numbers = gene counts). [file Image3.jpeg]
