# Supplementary material for: Deciphering intra-connectivity of gene network response to drought and salinity in apple
Source: Front Plant Sci. 2026 Mar 16;17:1763760. doi: 10.3389/fpls.2026.1763760 (PMC13033804; doi:10.3389/fpls.2026.1763760)
Supplement: Supplementary file 4 [file Table1.doc]

**Supplementary Table 1. Sequencing reads and reads mapping of RNA-sequencing**

| **Number** | **Treament** | **Stages** | **Replicate** | **Clean reads** | **Unique mapped reads** | **Unique mapped ratio** | **Mapped reads** | **Mapped ratio** |
| --- | --- | --- | --- | --- | --- | --- | --- | --- |
| 1 | Control | 0h | replicate 1 | 17199359 | 15115695 | 87.89% | 16243075 | 94.44% |
| 2 | Control | 0h | replicate 2 | 17503981 | 15480163 | 88.44% | 16499252 | 94.26% |
| 3 | Control | 0h | replicate 3 | 17397700 | 15349008 | 88.22% | 16399072 | 94.26% |
| 4 | NaCl | 1h | replicate 1 | 17318522 | 15262088 | 88.13% | 16371199 | 94.53% |
| 5 | NaCl | 1h | replicate 2 | 18126234 | 15922583 | 87.84% | 17096664 | 94.32% |
| 6 | NaCl | 1h | replicate 3 | 17341364 | 15286637 | 88.15% | 16401462 | 94.58% |
| 7 | NaCl | 6h | replicate 1 | 17445209 | 15297347 | 87.69% | 16475255 | 94.44% |
| 8 | NaCl | 6h | replicate 2 | 16832432 | 14832384 | 88.12% | 15933580 | 94.66% |
| 9 | NaCl | 6h | replicate 3 | 17557399 | 15397402 | 87.70% | 16598765 | 94.54% |
| 10 | NaCl | 12h | replicate 1 | 17455038 | 15344329 | 87.91% | 16549122 | 94.81% |
| 11 | NaCl | 12h | replicate 2 | 18265400 | 16023039 | 87.72% | 17257150 | 94.48% |
| 12 | NaCl | 12h | replicate 3 | 17706753 | 15510095 | 87.59% | 16741735 | 94.55% |
| 13 | NaCl | 24h | replicate 1 | 17493086 | 15386921 | 87.96% | 16537964 | 94.54% |
| 14 | NaCl | 24h | replicate 2 | 17603944 | 15431584 | 87.66% | 16637487 | 94.51% |
| 15 | NaCl | 24h | replicate 3 | 18228015 | 15768927 | 86.51% | 17134334 | 94.00% |
| 16 | PEG | 1h | replicate 1 | 17599276 | 15459827 | 87.84% | 16615476 | 94.41% |
| 17 | PEG | 1h | replicate 2 | 17367158 | 15163863 | 87.31% | 16330339 | 94.03% |
| 18 | PEG | 1h | replicate 3 | 17491615 | 15263335 | 87.26% | 16393142 | 93.72% |
| 19 | PEG | 6h | replicate 1 | 18220013 | 16019869 | 87.92% | 17252530 | 94.69% |
| 20 | PEG | 6h | replicate 2 | 17566159 | 15315565 | 87.19% | 16543809 | 94.18% |
| 21 | PEG | 6h | replicate 3 | 17805540 | 15700526 | 88.18% | 16808430 | 94.40% |
| 22 | PEG | 12h | replicate 1 | 17675543 | 15484277 | 87.60% | 16685713 | 94.40% |
| 23 | PEG | 12h | replicate 2 | 17526258 | 15382137 | 87.77% | 16530767 | 94.32% |
| 24 | PEG | 12h | replicate 3 | 18241263 | 15967036 | 87.53% | 17208808 | 94.34% |
| 25 | PEG | 24h | replicate 1 | 17362822 | 15141120 | 87.20% | 16322789 | 94.01% |
| 26 | PEG | 24h | replicate 2 | 17253889 | 15168722 | 87.91% | 16315277 | 94.56% |
| 27 | PEG | 24h | replicate 3 | 17869858 | 15653129 | 87.60% | 16835193 | 94.21% |
| Sum |  |  |  | 475453830 | 417127608 | 87.73% | 448718388 | 94.38% |
