# Supplementary material for: Deciphering intra-connectivity of gene network response to drought and salinity in apple
Source: Front Plant Sci. 2026 Mar 16;17:1763760. doi: 10.3389/fpls.2026.1763760 (PMC13033804; doi:10.3389/fpls.2026.1763760)
Supplement: Supplementary file 5 [file Table2.doc]

**Supplementary Table 2. Gene expression distribution of all the assembled genes from RNA-sequencing**

| **Layer** | **FPKM** | **CK_0** | **NaCl_1** | **NaCl_6** | **NaCl_12** | **NaCl_24** | **PEG_1** | **PEG_6** | **PEG_12** | **PEG_24** | **Average** | **Accumulative gene number of AGE** | **Accumulative density of AGE** | **proportion of AGE** |
| --- | --- | --- | --- | --- | --- | --- | --- | --- | --- | --- | --- | --- | --- | --- |
| **Layer0** | 0 | 8195 | 8146 | 7328 | 8412 | 8678 | 7702 | 7398 | 8234 | 7578 | 3642 | 3642 | 0.07822501 | **0.07822501** |
| Layer1 | (0, 1] | 10981 | 11613 | 11584 | 11732 | 12016 | 11418 | 11492 | 10436 | 11399 | 15142 | 15142 | 0.325228747 | 0.325228747 |
| Layer2 | (1, 2] | 3557 | 3762 | 3554 | 3699 | 3545 | 3681 | 3634 | 3625 | 3637 | 3576 | 3576 | 0.076807423 | 0.076807423 |
| Layer3 | (2, 3] | 2610 | 2584 | 2436 | 2535 | 2452 | 2587 | 2429 | 2517 | 2470 | 2591 | 2591 | 0.055651016 | 0.055651016 |
| Layer4 | (3, 4] | 2097 | 2066 | 1914 | 1952 | 1884 | 2067 | 2055 | 2103 | 2090 | 1975 | 1975 | 0.042420207 | 0.042420207 |
| Layer5 | (4, 5] | 1764 | 1681 | 1701 | 1671 | 1598 | 1662 | 1625 | 1687 | 1736 | 1723 | 1723 | 0.037007603 | 0.037007603 |
| Layer6 | (5, 6] | 1483 | 1498 | 1473 | 1307 | 1354 | 1559 | 1509 | 1508 | 1453 | 1509 | 1509 | 0.032411186 | 0.032411186 |
| Layer7 | (6, 7] | 1348 | 1243 | 1257 | 1186 | 1178 | 1316 | 1328 | 1283 | 1322 | 1357 | 1357 | 0.029146441 | 0.029146441 |
| Layer8 | (7, 8] | 1179 | 1093 | 1162 | 1090 | 982 | 1124 | 1193 | 1182 | 1157 | 1119 | 1119 | 0.024034538 | 0.024034538 |
| Layer9 | (8, 9] | 992 | 986 | 1006 | 878 | 884 | 1027 | 927 | 1037 | 1006 | 1015 | 1015 | 0.021800765 | 0.021800765 |
| Layer10 | (9, 10] | 836 | 808 | 894 | 860 | 824 | 843 | 927 | 879 | 852 | 868 | 868 | 0.018643413 | 0.018643413 |
| Layer11 | (10, 50] | 9287 | 8638 | 9891 | 8943 | 8852 | 9196 | 9655 | 9744 | 9613 | 9679 | 9679 | 0.207891232 | 0.207891232 |
| Layer12 | (50, ꝏ) | 2229 | 2440 | 2358 | 2293 | 2311 | 2376 | 2386 | 2323 | 2245 | 2362 | 2362 | 0.05073242 | 0.05073242 |
| **Note:AGE means average genes' expression** | | | | | | | | | | | | | | |
