# Supplementary material for: Deciphering intra-connectivity of gene network response to drought and salinity in apple
Source: Front Plant Sci. 2026 Mar 16;17:1763760. doi: 10.3389/fpls.2026.1763760 (PMC13033804; doi:10.3389/fpls.2026.1763760)
Supplement: Supplementary file 6 [file Table3.doc]

**Supplementary Table 3. Statistics of gene number under different comparison**

| **Comparison groups** | **Gene Number** | **Union of 20 gourps** | **Union of 10 groups** | **Intersection of 10 groups** | **Union of CK vs Treatment groups** | **Intersection of CK vs Treatment groups** | **Down-regulated** | **Up-regulated** |
| --- | --- | --- | --- | --- | --- | --- | --- | --- |
| **CK vs NaCl_1** | 3691 | 18707 | 17508 | 4 | 12144 | **1526** | 929 | 542 |
| **CK vs NaCl_6** | 5405 |  |  |  |  |  |  |  |
| **CK vs NaCl_12** | 9087 |  |  |  |  |  |  |  |
| **CK vs NaCl_24** | 7082 |  |  |  |  |  |  |  |
| NaCl_1 vs NaCl_6 | 4465 |  |  |  |  |  |  |  |
| NaCl_1 vs NaCl_12 | 8532 |  |  |  |  |  |  |  |
| NaCl_1 vs NaCl_24 | 9902 |  |  |  |  |  |  |  |
| NaCl_6 vs NaCl_12 | 3959 |  |  |  |  |  |  |  |
| NaCl_6 vs NaCl_24 | 5715 |  |  |  |  |  |  |  |
| NaCl_12 vs NaCl_24 | 1273 |  |  |  |  |  |  |  |
| **CK vs PEG_1** | 3819 |  | 11303 | 0 | 7506 | **712** | 514 | 186 |
| **CK vs PEG_6** | 1550 |  |  |  |  |  |  |  |
| **CK vs PEG_12** | 2772 |  |  |  |  |  |  |  |
| **CK vs PEG_24** | 4971 |  |  |  |  |  |  |  |
| PEG_1 vs PEG_6 | 2431 |  |  |  |  |  |  |  |
| PEG_1 vs PEG_12 | 3758 |  |  |  |  |  |  |  |
| PEG_1 vs PEG_24 | 6396 |  |  |  |  |  |  |  |
| PEG_6 vs PEG_12 | 539 |  |  |  |  |  |  |  |
| PEG_6 vs PEG_24 | 2315 |  |  |  |  |  |  |  |
| PEG_12 vs PEG_24 | 1372 |  |  |  |  |  |  |  |
