# Supplementary material for: Deciphering intra-connectivity of gene network response to drought and salinity in apple
Source: Front Plant Sci. 2026 Mar 16;17:1763760. doi: 10.3389/fpls.2026.1763760 (PMC13033804; doi:10.3389/fpls.2026.1763760)
Supplement: Supplementary file 7 [file Table4.doc]

**Supplementary Table 4. Summary of top hub TFs/TRs genes in co-expression module MEpink and MEred**

| **Gene ID** | **KME** | **Module** | **DEGs** | **Family** | **CK** | **NaCl_1** | **NaCl_6** | **NaCl_12** | **NaCl_24** | **PEG_1** | **PEG_6** | **PEG_12** | **PEG_24** |
| --- | --- | --- | --- | --- | --- | --- | --- | --- | --- | --- | --- | --- | --- |
| MD08G1096000 | 0.958059182 | pink | nonDEG | ERF | 11.43 | 7.37 | 24.74 | 28.80 | 34.43 | 9.55 | 23.01 | 22.40 | 30.39 |
| MD12G1131800 | 0.946278965 | pink | nonDEG | RWP-RK | 6.18 | 45.41 | 5.74 | 1.71 | 0.46 | 56.54 | 8.26 | 10.64 | 2.80 |
| MD05G1011200 | 0.920737201 | pink | nonDEG | GRAS | 4.86 | 12.64 | 2.58 | 0.61 | 0.44 | 15.48 | 4.36 | 4.48 | 0.94 |
| MD02G1156700 | 0.905929999 | pink | nonDEG | LIM | 15.44 | 6.07 | 14.16 | 21.10 | 23.81 | 8.61 | 16.40 | 17.62 | 16.83 |
| MD17G1112600 | 0.901777208 | pink | nonDEG | WRKY | 18.99 | 27.77 | 20.78 | 12.75 | 14.82 | 31.69 | 19.62 | 20.03 | 18.87 |
| MD15G1217100 | 0.887105706 | pink | nonDEG | GNAT | 42.08 | 90.70 | 42.12 | 11.40 | 10.85 | 66.04 | 45.40 | 38.33 | 28.98 |
| MD15G1213400 | 0.886358324 | pink | nonDEG | NF-YA | 16.42 | 18.41 | 19.28 | 35.15 | 41.06 | 15.98 | 23.95 | 24.29 | 31.25 |
| MD10G1051700 | 0.876021454 | pink | nonDEG | CAMTA | 4.26 | 15.56 | 8.59 | 3.37 | 2.04 | 14.85 | 6.38 | 6.09 | 3.67 |
| MD02G1086200 | 0.874279463 | pink | nonDEG | NF-YA | 13.90 | 22.29 | 15.96 | 10.77 | 11.26 | 23.86 | 14.57 | 15.38 | 11.83 |
| MD07G1297100 | 0.863256957 | pink | DEG | HB | **23.31** | **1.86** | 17.45 | 38.02 | **76.09** | **3.15** | 28.70 | 32.09 | 55.67 |
| MD13G1077900 | 0.859624702 | pink | nonDEG | WRKY | 9.53 | 20.41 | 9.22 | 7.48 | 7.34 | 18.20 | 10.37 | 12.35 | 11.21 |
| MD07G1222500 | 0.937497328 | red | nonDEG | Alfin-like | 5.79 | 15.25 | 4.44 | 2.24 | 0.99 | 25.29 | 5.92 | 8.45 | 2.83 |
| MD17G1189100 | 0.928935955 | red | nonDEG | AUX/IAA | 39.19 | 24.84 | 49.68 | 74.01 | 97.05 | 22.17 | 45.02 | 40.71 | 72.14 |
| MD14G1149600 | 0.923714551 | red | DEG | bHLH | **32.91** | **87.60** | 25.45 | 15.42 | **13.22** | **125.86** | 42.18 | 48.29 | 15.21 |
| MD03G1119800 | 0.919746126 | red | nonDEG | C2C2-GATA | 29.57 | 27.05 | 31.97 | 56.89 | 49.00 | 25.78 | 27.96 | 32.44 | 50.49 |
| MD03G1262500 | 0.91935878 | red | nonDEG | OFP | 22.70 | 15.38 | 20.91 | 33.78 | 36.21 | 16.34 | 19.06 | 21.62 | 27.84 |
| MD06G1178800 | 0.902315454 | red | nonDEG | LIM | 13.81 | 81.88 | 20.00 | 13.18 | 14.35 | 75.48 | 13.87 | 18.32 | 19.90 |
| MD09G1184000 | 0.894387555 | red | DEG | MYB | **9.05** | **1.42** | 3.16 | 10.96 | **17.18** | **1.70** | 4.98 | 4.77 | 7.29 |
| MD08G1016200 | 0.889835788 | red | nonDEG | bZIP | 11.23 | 33.36 | 15.80 | 9.33 | 4.50 | 32.14 | 13.86 | 11.75 | 7.80 |
| MD15G1021800 | 0.889572215 | red | nonDEG | HB | 3.47 | 15.31 | 7.05 | 3.53 | 3.91 | 16.38 | 4.13 | 3.89 | 5.46 |
| MD02G1087900 | 0.875417281 | red | DEG | MYB | **12.00** | **6.47** | 26.97 | 35.85 | **43.32** | **8.26** | 21.70 | 22.04 | 49.82 |
| MD02G1087700 | 0.871019515 | red | nonDEG | MYB | 14.95 | 8.63 | 16.00 | 21.79 | 19.78 | 10.52 | 14.62 | 14.74 | 19.01 |
| MD15G1300200 | 0.869455407 | red | nonDEG | bZIP | 9.90 | 36.61 | 10.49 | 7.43 | 3.45 | 37.50 | 10.17 | 13.96 | 9.45 |
| MD16G1020800 | 0.868069982 | red | nonDEG | Tify | 15.39 | 58.28 | 30.24 | 18.45 | 14.37 | 54.45 | 16.71 | 27.26 | 16.73 |
| MD08G1070700 | 0.853069285 | red | DEG | MYB | **29.16** | **22.78** | 28.41 | 34.68 | **37.58** | **25.98** | 26.53 | 26.31 | 35.96 |
| MD13G1030000 | 0.852312673 | red | nonDEG | Tify | 20.86 | 53.13 | 22.50 | 6.73 | 10.44 | 57.27 | 30.06 | 25.38 | 10.53 |
