# Supplementary material for: Deciphering intra-connectivity of gene network response to drought and salinity in apple
Source: Front Plant Sci. 2026 Mar 16;17:1763760. doi: 10.3389/fpls.2026.1763760 (PMC13033804; doi:10.3389/fpls.2026.1763760)
Supplement: Supplementary file 8 [file Table5.doc]

**Supplementary Table 5. Transcriptomic profiling of genes involved in plant hormone signal transduction**

| **Gene ID** | **Gene Name** | **Gene Annotion** | **CK_0** | **NaCl_1** | **NaCl_6** | **NaCl_12** | **NaCl_24** | **PEG_1** | **PEG_6** | **PEG_12** | **PEG_24** |
| --- | --- | --- | --- | --- | --- | --- | --- | --- | --- | --- | --- |
| MD17G1132600 | *MdPIL6* | phytochrome interacting factor 3-like 6 | 35.56256867 | 18.805004 | 33.415956 | 40.933698 | 26.44513333 | 23.18788467 | 22.72289467 | 59.521773 | 50.80663333 |
| MD15G1077100 | *MdCYCD3;1* | CYCLIN D3;1 | 8.390156667 | 6.857318 | 18.16032933 | 6.383322667 | 2.314837 | 9.517360667 | 11.92315133 | 10.856762 | 13.81170433 |
| MD13G1237300 | *MdXTR6* | xyloglucan endotransglycosylase 6 | 19.89434467 | 335.9030153 | 32.902937 | 20.73155967 | 24.037887 | 210.5830943 | 55.21694433 | 39.81026833 | 34.57948267 |
| MD09G1203100 | *MdAHP4* | HPT phosphotransmitter 4 | 0.085690333 | 0.15489 | 0.07907 | 0.009393333 | 0.031886333 | 0.512016333 | 0.161924 | 0.244534333 | 0.031185333 |
| MD09G1091000 | *MdDFL2* | Auxin-responsive GH3 family protein | 20.423097 | 4.890822667 | 7.900730667 | 1.742359 | 1.109924333 | 6.402123333 | 12.21118667 | 20.89668033 | 5.456355333 |
| MD09G1089100 | *MdSAUR* | SAUR-like auxin-responsive protein family | 0 | 0 | 0 | 0 | 0.124432333 | 0.043383 | 0.081321667 | 0.043055667 | 0.128179667 |
| MD08G1221700 | *MdBAK1* | BRI1-associated receptor kinase | 15.90646767 | 37.06771433 | 26.62761433 | 32.807712 | 22.024372 | 35.30684367 | 24.46520133 | 19.436758 | 16.71004867 |
| MD08G1133700 | *MdCAP* | CAP superfamily protein | 0 | 0 | 0 | 0 | 0.124432333 | 0.043383 | 0.081321667 | 0.043055667 | 0.128179667 |
| MD07G1047300 | *MdTGA6* | TGACG motif-binding factor 6 | 10.50633267 | 7.642481333 | 15.048912 | 12.741656 | 11.89410033 | 8.745845667 | 11.96933233 | 13.72448333 | 16.08554567 |
| MD05G1309400 | *MdETT* | Transcriptional factor B3 family protein | 0.887581 | 0.815064667 | 0.880296 | 0.715635 | 0.783282 | 0.785635 | 1.098149333 | 1.521106 | 1.156206 |
| MD05G1256300 | *MdNPR3-1* | NPR1-like protein 3 | 2.272653667 | 8.983663 | 5.514538667 | 10.85963567 | 7.965316333 | 7.668668333 | 4.363843667 | 5.849668 | 6.232190667 |
| MD04G1225000 | *MdNPR3-2* | AUX/IAA transcriptional regulator family protein | 109.9092483 | 38.25345767 | 109.0656943 | 78.917015 | 49.09770067 | 32.934926 | 88.32118 | 77.28140133 | 69.574637 |
| MD02G1266800 | *MdAREB3* | ABA-responsive element binding protein 3 | 6.796512 | 6.325286667 | 11.15415233 | 14.58670833 | 12.27219433 | 7.000495333 | 9.510053 | 12.71455233 | 11.70743767 |
| MD02G1166500 | *MdSNF1* | sucrose nonfermenting 1(SNF1)-related protein kinase 2.3 | 67.39074467 | 32.277938 | 90.24595867 | 125.49632 | 131.056877 | 36.801071 | 73.01141067 | 80.99525733 | 158.2247313 |
| MD02G1096100 | *MdJAZ1* | jasmonate-zim-domain protein 1 | 113.9102987 | 1225.480428 | 445.6029153 | 239.7156267 | 219.982966 | 1067.819112 | 253.9595133 | 263.296636 | 304.6251627 |
| MD01G1147000 | *MdCOI1* | RNI-like superfamily protein | 57.015231 | 20.14139633 | 40.06642933 | 48.44231033 | 61.94007733 | 22.17281867 | 39.716989 | 43.85536733 | 67.55006133 |
| MD01G1139200 | *MdPP2CA* | protein phosphatase 2CA | 18.683376 | 25.686955 | 12.216189 | 10.04090933 | 15.933222 | 19.27875933 | 11.24076067 | 11.31790233 | 36.074619 |
| MD01G1078900 | *MdRCAR1* | regulatory component of ABA receptor 1 | 130.9477743 | 74.910736 | 129.0845363 | 141.223124 | 162.1724143 | 80.062627 | 121.6377513 | 103.0626093 | 149.8717043 |
