# Supplementary material for: Deciphering intra-connectivity of gene network response to drought and salinity in apple
Source: Front Plant Sci. 2026 Mar 16;17:1763760. doi: 10.3389/fpls.2026.1763760 (PMC13033804; doi:10.3389/fpls.2026.1763760)
Supplement: Supplementary file 9 [file Table6.doc]

**Supplementary Table 6. Transcriptomic profiling of genes involved in photosynthesis**

| **Gnen ID** | **Gene Name** | **Gene Anotation** | **CK_0** | **NaCl_1** | **NaCl_6** | **NaCl_12** | **NaCl_24** | **PEG_1** | **PEG_6** | **PEG_12** | **PEG_24** |
| --- | --- | --- | --- | --- | --- | --- | --- | --- | --- | --- | --- |
| MD17G1209900 | *MdPSAO* | photosystem I subunit O | 0 | 0 | 0 | 0 | 0.124432333 | 0.043383 | 0.081321667 | 0.043055667 | 0.128179667 |
| MD17G1132900 | *MdPSBP-1* | photosystem II subunit P-1 | 284.411611 | 224.6369577 | 150.1891633 | 127.4395853 | 142.082606 | 182.1817527 | 199.01887 | 250.3714497 | 211.350174 |
| MD17G1079700 | *MdPSB27* | photosystem II family protein | 75.214924 | 19.37331533 | 45.96769833 | 18.60555067 | 21.027291 | 26.80905333 | 40.01728967 | 43.25697467 | 35.79197433 |
| MD15G1441800 | *MdPSAN* | chloroplast, putative / PSI-N, putative (PSAN) | 139.1715903 | 74.27340333 | 75.849218 | 80.792511 | 71.75782667 | 60.444776 | 94.13396067 | 146.318171 | 86.48262267 |
| MD15G1428700 | *Md2Fe-2S* | 2Fe-2S ferredoxin-like superfamily protein | 962.093058 | 841.0750327 | 617.0503947 | 529.3829143 | 447.8284097 | 742.7277427 | 807.8700563 | 945.5948893 | 804.1877847 |
| MD15G1272500 | *MdPSBO2* | photosystem II subunit O-2 | 586.6486713 | 267.150757 | 248.2094063 | 196.2446797 | 215.7049157 | 260.9042107 | 300.7727203 | 338.9894003 | 263.9945473 |
| MD15G1126100 | *MdATPF* | ATPase, F0 complex, subunit B/B' | 125.7727293 | 72.97792067 | 31.897478 | 24.75838467 | 26.282411 | 49.12211967 | 45.42491667 | 55.217317 | 26.90932267 |
| MD15G1006300 | *MdPETE1* | plastocyanin 1 | 2497.673665 | 795.707601 | 629.2309163 | 550.7322287 | 484.1637877 | 732.0051473 | 1034.391235 | 1028.013509 | 561.412781 |
| MD13G1191300 | *MdPSAD-2* | photosystem I subunit D-2 | 1401.22701 | 521.7879233 | 666.6244507 | 924.3146157 | 713.832906 | 440.6840007 | 795.5062357 | 869.8917847 | 629.6428017 |
| MD13G1106700 | *MdPSBY* | photosystem II BY | 1227.743266 | 350.7459613 | 385.7868957 | 367.0913797 | 400.032969 | 440.683899 | 639.5539147 | 797.2009277 | 310.543335 |
| MD11G1068500 | *MdPSAA* | Photosystem I, PsaA/PsaB protein | 12.43894233 | 6.359071 | 4.219132667 | 3.783687 | 5.725763333 | 8.932823 | 12.37637567 | 4.607927333 | 3.149849 |
| MD10G1192100 | *MdPSBW* | photosystem II reaction center W | 230.304769 | 102.0905 | 140.6483867 | 100.3749593 | 106.461984 | 81.366432 | 138.6682587 | 183.3579813 | 126.56219 |
| MD10G1043200 | *MdATPD* | ATP synthase delta-subunit gene | 285.2837677 | 85.71819533 | 152.6381787 | 105.031006 | 126.3834863 | 96.586098 | 183.0473633 | 190.667816 | 136.1298193 |
| MD09G1209500 | *MdPSAH2* | photosystem I subunit H2 | 491.2984823 | 242.8366443 | 164.051473 | 95.00872033 | 120.123296 | 183.3804473 | 182.648506 | 259.4969583 | 165.1236113 |
| MD09G1098300 | *MdPETC* | photosynthetic electron transfer C | 253.354472 | 79.26413233 | 47.24510967 | 37.90793567 | 44.67871133 | 75.22280133 | 72.23657467 | 79.05214967 | 72.46404367 |
| MD08G1162600 | *MdLIL3:1* | Chlorophyll A-B binding family protein | 336.359558 | 221.453491 | 50.90676133 | 17.59780367 | 24.502152 | 170.099884 | 102.0451253 | 82.98921233 | 38.71013133 |
| MD05G1347300 | *MdATPC1* | ATPase, F1 complex, gamma subunit protein | 476.5018057 | 133.7647247 | 134.323331 | 81.18320567 | 95.577779 | 157.2030383 | 230.900533 | 159.69311 | 81.917895 |
| MD05G1215200 | *MdPSBR* | photosystem II subunit R | 891.278544 | 400.987732 | 335.279378 | 600.166524 | 831.6055703 | 291.3495177 | 395.3596697 | 843.4303183 | 1397.287252 |
| MD04G1244400 | *MdPSAL* | photosystem I subunit l | 781.25649 | 380.6805827 | 185.1336213 | 77.29324333 | 88.45635233 | 318.3070577 | 251.930776 | 229.107951 | 124.102033 |
| MD03G1212800 | *MdPQL1* | PsbQ-like 1 | 13.234001 | 4.245404 | 4.665065 | 2.278974333 | 1.712862 | 4.466844333 | 7.602993667 | 6.337757667 | 5.322422 |
| MD03G1042800 | *MdPSAG* | photosystem I subunit G | 1011.8031 | 330.1507823 | 384.6376547 | 225.430781 | 218.9259337 | 311.1229757 | 433.7075297 | 415.7354027 | 299.6752727 |
| MD02G1130800 | *MdFNR1* | ferredoxin-NADP(+)-oxidoreductase 1 | 385.7960207 | 112.858503 | 144.90508 | 113.4799907 | 122.7234037 | 119.651787 | 176.840515 | 163.6145273 | 162.618688 |
| MD00G1109700 | *MdPSAK* | photosystem I subunit K | 670.272939 | 320.9287823 | 271.0842287 | 179.0920513 | 127.420001 | 322.5513763 | 344.0145823 | 449.4113263 | 150.010887 |
