# Supplementary material for: Deciphering intra-connectivity of gene network response to drought and salinity in apple
Source: Front Plant Sci. 2026 Mar 16;17:1763760. doi: 10.3389/fpls.2026.1763760 (PMC13033804; doi:10.3389/fpls.2026.1763760)
Supplement: Supplementary file 10 [file Table7.doc]

| **Gene ID** | **Gene Name** | **Gene Annotation** | **CK_0** | **NaCl_1** | **NaCl_6** | **NaCl_12** | **NaCl_24** | **PEG_1** | **PEG_6** | **PEG_12** | **PEG_24** |
| --- | --- | --- | --- | --- | --- | --- | --- | --- | --- | --- | --- |
| MD03G1138100 | *MdNRS/ER* | nucleotide-rhamnose synthase/epimerase-reductase | 0.284205 | 0.354392 | 0.173007333 | 0.08945 | 0.077358 | 0.154474333 | 0.236538667 | 0.226563333 | 0.190386 |
| MD04G1224100 | *MdGME* | GDP-D-mannose 3',5'-epimerase | 15.762996 | 2.884333 | 2.147145667 | 1.741617667 | 3.043461667 | 2.414107 | 3.280976 | 5.402990667 | 7.831813 |
| MD05G1099800 | *MdNADP1* | NAD(P)-binding Rossmann-fold superfamily protein | 48.131893 | 53.645177 | 39.58788967 | 22.671228 | 24.40300933 | 47.995256 | 44.07622667 | 36.51396867 | 24.94089267 |
| MD05G1248900 | *MdGlcNAc1pUT1* | N-acetylglucosamine-1-phosphate uridylyltransferase 1 | 6.146249667 | 3.996102 | 3.063595 | 1.576012333 | 1.548624667 | 4.074777333 | 3.738280333 | 4.399744 | 2.670086333 |
| MD06G1200200 | *MdUDP6* | UDP-glucose 6-dehydrogenase family protein | 1.52139 | 3.133687333 | 1.867911 | 0.702637 | 0.652334 | 2.70989 | 1.304185 | 1.712228333 | 1.272247 |
| MD08G1027900 | *MdAPL2* | ADPGLC-PPase large subunit | 3.033361667 | 2.567446667 | 3.407776333 | 1.259146667 | 1.219348 | 2.752381 | 2.808559 | 3.648036333 | 6.749711 |
| MD09G1023000 | *MdUXS6* | glucuronokinase G | 0.151758333 | 0.134034 | 0.594331333 | 0.514774 | 0.521362 | 0.124549667 | 0.217409 | 0.360742 | 0.75141 |
| MD11G1143300 | *MdUXS6-1* | UDP-XYL synthase 6 | 15.37685767 | 22.01868367 | 14.73272533 | 9.727948333 | 11.147384 | 19.102373 | 15.51064333 | 15.98954067 | 11.870362 |
| MD11G1154500 | *MdAXS2* | UDP-D-apiose/UDP-D-xylose synthase 2 | 62.10524133 | 89.64904033 | 55.229318 | 37.73225933 | 31.71222433 | 86.35289233 | 59.57437133 | 65.97697933 | 43.65033833 |
| MD12G1112100 | *MdASD1* | alpha-L-arabinofuranosidase 1 | 9.692709 | 7.849020333 | 8.318430333 | 11.76883133 | 12.449232 | 6.385694 | 9.546647 | 11.11966767 | 11.19671733 |
| MD13G1235800 | *MdGalAK* | galacturonic acid kinase | 3.102049667 | 3.579632667 | 2.556406 | 1.156095667 | 0.671429667 | 3.568234667 | 2.427106 | 2.660361333 | 1.516852 |
| MD13G1282100 | *MdNDS* | Nucleotide-diphospho-sugar transferases superfamily protein | 0.005456 | 0.021590333 | 0 | 0 | 0.124432333 | 0.043383 | 0.081321667 | 0.043055667 | 0.128179667 |
| MD14G1129500 | *MdUGE5* | UDP-D-glucose/UDP-D-galactose 4-epimerase 5 | 9.763785 | 5.800339 | 9.461792333 | 7.714951667 | 8.737853667 | 7.398576 | 8.969739 | 8.502759 | 8.238558333 |
| MD14G1187500 | *MdAGPP* | ADP-glucose pyrophosphorylase family protein | 11.00108167 | 6.485971 | 12.363836 | 7.213818667 | 5.652366667 | 6.707302 | 9.799619667 | 12.582157 | 9.925155667 |
| MD15G1031400 | *MdSIS* | Sugar isomerase (SIS) family protein | 20.21746533 | 14.96359167 | 13.45482867 | 13.37538633 | 16.42421933 | 12.62208667 | 15.41784733 | 15.734935 | 16.03258333 |
| MD15G1247100 | *MdNADP2* | NAD(P)-binding Rossmann-fold superfamily protein | 74.51463567 | 296.064402 | 60.855221 | 43.67872233 | 42.22414767 | 183.0653203 | 72.76107033 | 50.28562933 | 38.447665 |
| MD15G1414100 | *MdXYL4* | beta-D-xylosidase 4 | 3.385773 | 3.029096 | 0.865156 | 0.365625333 | 0.079581667 | 3.028297333 | 1.273745 | 1.564639333 | 0.504833667 |
| MD16G1022300 | *MdPGM* | Phosphoglucomutase/phosphomannomutase family protein | 61.23444233 | 77.22997767 | 88.44128433 | 76.508222 | 85.75389867 | 82.10568733 | 86.44991433 | 71.255567 | 65.22876633 |
| MD16G1260200 | *MdGAE1* | UDP-D-glucuronate 4-epimerase 1 | 6.076895667 | 10.309875 | 5.799629667 | 4.499278 | 3.394766667 | 8.662096667 | 6.751792 | 7.336911 | 6.961598667 |
| MD17G1127400 | *MdNADP3* | NAD(P)-binding Rossmann-fold superfamily protein | 0.388417667 | 0.506035 | 0.377019667 | 0.191723667 | 0.084706667 | 0.700983 | 0.297647333 | 0.474821667 | 0.295486333 |
| MD17G1140800 | *MdRHM1* | rhamnose biosynthesis 1 | 5.001382333 | 2.481243667 | 2.024778333 | 0.847554667 | 0.526600667 | 2.541735667 | 2.240970667 | 2.644465667 | 1.392894333 |

**Supplementary Table 7. Transcriptomic profiling of genes involved in amino sugar and nucleotide sugar metabolism**
