# Supplementary material for: Deciphering intra-connectivity of gene network response to drought and salinity in apple
Source: Front Plant Sci. 2026 Mar 16;17:1763760. doi: 10.3389/fpls.2026.1763760 (PMC13033804; doi:10.3389/fpls.2026.1763760)
Supplement: Supplementary file 11 [file Table8.doc]

| **Gene ID** | **Gene Name** | **Gene Annotation** | **CK_0** | **NaCl_1** | **NaCl_6** | **NaCl_12** | **NaCl_24** | **PEG_1** | **PEG_6** | **PEG_12** | **PEG_24** |
| --- | --- | --- | --- | --- | --- | --- | --- | --- | --- | --- | --- |
| MD01G1238700 | *MdDEH* | GroES-like zinc-binding dehydrogenase family protein | 3.014332667 | 2.321374 | 7.479145 | 31.74824833 | 19.36424933 | 5.194107333 | 12.66958333 | 9.788330667 | 11.31159167 |
| MD02G1009300 | *MdALDH11A3* | aldehyde dehydrogenase 11A3 | 137.9792303 | 58.76358133 | 38.10624133 | 43.67258833 | 52.19471733 | 51.33803433 | 53.52863033 | 75.13472267 | 31.745135 |
| MD04G1109800 | *MdPCK2* | phosphoenolpyruvate carboxykinase 2 | 1.191835667 | 0.855026667 | 1.307153 | 0.843162667 | 0.718341333 | 1.291925333 | 1.300580333 | 1.389353333 | 1.169461 |
| MD05G1013200 | *MdADH1* | alcohol dehydrogenase 1 | 0.392401667 | 0.288032333 | 1.074215 | 0.734982333 | 0.211422 | 1.046153 | 8.678472 | 0.349836333 | 0.33162 |
| MD09G1202200 | *MdHXK2* | hexokinase 2 | 3.086462667 | 2.039076667 | 2.840486 | 1.625291 | 2.091726667 | 2.698283 | 3.054718333 | 2.609757333 | 2.103961333 |
| MD10G1063600 | *MdFBA2* | fructose-bisphosphate aldolase 2 | 338.6939597 | 243.3891753 | 87.34203833 | 57.96021033 | 53.229913 | 205.6291453 | 140.975268 | 217.813619 | 63.698475 |
| MD10G1118200 | *MdTPI* | triosephosphate isomerase | 77.87103767 | 61.88979833 | 46.650842 | 37.41776033 | 40.83897133 | 65.78325 | 55.64905833 | 46.881921 | 40.09962067 |
| MD10G1210500 | *MdPGK* | Phosphoglycerate kinase family protein | 249.825592 | 166.918035 | 91.15952567 | 34.01449767 | 37.06229267 | 143.08342 | 124.799873 | 92.16024767 | 42.21074167 |
| MD10G1210900 | *MdGAPCP-1* | glyceraldehyde-3-phosphate dehydrogenase of plastid 1 | 7.844780667 | 15.923446 | 12.65847767 | 3.571079333 | 4.463024 | 20.38598933 | 12.41329933 | 8.175872 | 5.595153667 |
| MD12G1149800 | *MdATB2* | NAD(P)-linked oxidoreductase superfamily protein | 1.765691333 | 13.205997 | 56.75670767 | 86.51745333 | 54.17478067 | 11.47683233 | 17.072165 | 7.158854667 | 12.068778 |
| MD13G1090600 | *MdALDH2B7* | aldehyde dehydrogenase 2B7 | 23.59411667 | 23.042487 | 75.7169 | 70.24340567 | 56.53907133 | 26.08023833 | 55.08302033 | 50.02501267 | 55.157153 |
| MD13G1147400 | *MdLPD2* | lipoamide dehydrogenase 2 | 64.42309733 | 50.909316 | 44.57197567 | 38.20184533 | 32.982287 | 49.02106733 | 52.26622667 | 38.719551 | 31.321229 |
| MD15G1327600 | *MdPHM* | Phosphoglycerate mutase family protein | 0.686898 | 0.543509 | 0.921232667 | 0.679192 | 0.820882667 | 0.729488333 | 0.857193667 | 0.847585 | 1.102217333 |
| MD16G1010900 | *MdMLDH* | Lactate/malate dehydrogenase family protein | 3.724418333 | 5.701775667 | 8.614283 | 21.86780733 | 6.761283 | 4.994028667 | 17.042496 | 6.454546333 | 3.822437667 |
| MD17G1003300 | *MdACS* | acetyl-CoA synthetase | 8.554473333 | 20.291844 | 15.97431367 | 29.57359667 | 21.95946833 | 21.20088167 | 13.90792533 | 12.151996 | 16.10616867 |

**Supplementary Table 8. Transcriptomic profiling of genes involved in glycolysis / Gluconeogenesis**
