# Supplementary material for: Deciphering intra-connectivity of gene network response to drought and salinity in apple
Source: Front Plant Sci. 2026 Mar 16;17:1763760. doi: 10.3389/fpls.2026.1763760 (PMC13033804; doi:10.3389/fpls.2026.1763760)
Supplement: Supplementary file 12 [file Table9.doc]

**Supplementary Table 9. Transcriptomic profiling of genes involved in glyoxylate and dicarboxylate metabolism**

| **Gene ID** | **Gene Name** | **Gene Annotation** | **CK_0** | **NaCl_1** | **NaCl_6** | **NaCl_12** | **NaCl_24** | **PEG_1** | **PEG_6** | **PEG_12** | **PEG_24** |
| --- | --- | --- | --- | --- | --- | --- | --- | --- | --- | --- | --- |
| MD03G1168700 | *MdAGT* | alanine:glyoxylate aminotransferase | 636.9448547 | 131.976064 | 212.5328523 | 299.0727337 | 241.850332 | 125.126335 | 281.991491 | 308.8135987 | 235.54422 |
| MD06G1008600 | *MdCAT2-1* | catalase 2 | 171.8640773 | 79.18029767 | 102.6034697 | 121.4090603 | 143.8595353 | 87.43500767 | 133.054207 | 107.4446717 | 110.265803 |
| MD06G1008700 | *MdCAT2-2* | catalase 2 | 3911.164469 | 1468.456828 | 3806.598144 | 4083.184652 | 4706.532878 | 1467.213176 | 4382.590251 | 4482.670736 | 4917.811523 |
| MD08G1061400 | *MdGLDP2* | glycine decarboxylase P-protein 2 | 0.084544 | 0 | 0 | 0 | 0.124432333 | 0.043383 | 0.081321667 | 0.043055667 | 0.128179667 |
| MD09G1128400 | *MdKAT2* | Thiolase family protein | 40.45551167 | 292.913666 | 107.9520113 | 97.44840733 | 83.12966633 | 229.859675 | 81.292084 | 56.96235133 | 72.41439833 |
| MD09G1177800 | *MdDUSFL* | Aldolase-type TIM barrel family protein | 3.697103 | 4.153563 | 4.321929 | 4.189409667 | 9.056869 | 4.104982667 | 5.253788333 | 6.114866333 | 6.523691 |
| MD10G1323700 | *MdAMT* | Glycine cleavage T-protein family | 119.354406 | 80.721512 | 41.934985 | 18.15934233 | 22.65888967 | 70.27900933 | 61.178286 | 44.76414233 | 28.39156767 |
| MD10G1340000 | *MdPGLP2* | 2-phosphoglycolate phosphatase 2 | 7.333048667 | 7.767386333 | 8.750038333 | 7.402497 | 8.203 | 8.910656 | 8.60023 | 9.533659333 | 7.335609333 |
| MD11G1294900 | *MdRBCL* | ribulose-bisphosphate carboxylases | 6.990488 | 2.722658667 | 1.961071667 | 1.616840333 | 2.784127667 | 5.603472333 | 5.927639333 | 2.945385333 | 1.424872333 |
| MD13G1041800 | *MdRBCS1A* | ribulose bisphosphate carboxylase small chain 1A | 7.020523 | 2.810983667 | 7.174833667 | 2.393786 | 4.885267 | 2.843892667 | 5.582004 | 2.128047333 | 15.88819067 |
| MD13G1102800 | *MdAOAT2* | alanine-2-oxoglutarate aminotransferase 2 | 229.374842 | 84.26958467 | 50.55373033 | 64.54895767 | 60.77081267 | 73.876363 | 87.249013 | 81.290553 | 52.69249967 |
| MD14G1087500 | *MdGLU1* | glutamate synthase 1 | 194.106191 | 86.33788567 | 78.90414667 | 55.50591667 | 57.01393767 | 98.31197867 | 87.45677967 | 65.91543067 | 53.540615 |
| MD14G1161700 | *MdMLS* | malate synthase | 0 | 0 | 0 | 0 | 0.124432333 | 0.043383 | 0.081321667 | 0.043055667 | 0.128179667 |
| MD14G1238700 | *MdGSR1* | glutamine synthase clone R1 | 7.236616 | 18.40270333 | 27.18312 | 17.37838433 | 17.21357 | 22.30786267 | 17.40567333 | 24.062084 | 23.918609 |
| MD15G1307200 | *MdSHM1* | serine transhydroxymethyltransferase 1 | 181.9306767 | 73.870116 | 39.10372433 | 13.510784 | 10.08808267 | 64.22980867 | 54.03930067 | 46.87690733 | 21.794375 |
