# Supplementary material for: Deciphering intra-connectivity of gene network response to drought and salinity in apple
Source: Front Plant Sci. 2026 Mar 16;17:1763760. doi: 10.3389/fpls.2026.1763760 (PMC13033804; doi:10.3389/fpls.2026.1763760)
Supplement: Supplementary file 13 [file Table10.doc]

**Supplementary Table 10. Transcriptomic profiling of genes involved in starch and sucrose metabolism**

| **Gene** ID | **Gene Name** | **Gene Annotation** | **CK_0** | **NaCl_1** | **NaCl_6** | **NaCl_12** | **NaCl_24** | **PEG_1** | **PEG_6** | **PEG_12** | **PEG_24** |
| --- | --- | --- | --- | --- | --- | --- | --- | --- | --- | --- | --- |
| MD15G1319500 | *MdOGH17-1* | O-Glycosyl hydrolases family 17 protein | 23.96266167 | 13.07893067 | 27.23794433 | 14.34654967 | 15.63135767 | 19.82257433 | 25.347451 | 29.31577967 | 22.35433433 |
| MD15G1206200 | *MdALPL* | Alkaline-phosphatase-like family protein | 16.590981 | 25.67956533 | 19.00573933 | 31.69454067 | 43.95345967 | 33.98718967 | 24.657255 | 22.22123533 | 29.44733567 |
| MD14G1004200 | *MdXYL14-GH* | Glycosyl hydrolase family protein | 6.755843 | 4.611148333 | 8.312150333 | 11.36019633 | 15.39478467 | 6.359824667 | 11.87812567 | 6.935593 | 7.746077333 |
| MD13G1064200 | *MdBGLU40* | beta glucosidase 40 | 0.311891667 | 0.360224 | 0.201099 | 0.031639333 | 0 | 0.027755 | 0.009378667 | 0.087517667 | 0 |
| MD08G1027900 | *MdAPL2* | ADPGLC-PPase large subunit | 3.033361667 | 2.567446667 | 3.407776333 | 1.259146667 | 1.219348 | 2.752381 | 2.808559 | 3.648036333 | 6.749711 |
| MD06G1105900 | *MdGH9C2* | glycosyl hydrolase 9C2 | 47.495716 | 24.32120833 | 11.73055 | 11.32875533 | 4.234176667 | 29.70673767 | 11.70355433 | 10.65632033 | 6.874195667 |
| MD05G1342500 | *MdBGLU45* | beta-glucosidase 45 | 0.030320333 | 0 | 0 | 0 | 0.124432333 | 0.043383 | 0.081321667 | 0.043055667 | 0.128179667 |
| MD04G1001100 | *MdOGH17-2* | O-Glycosyl hydrolases family 17 protein | 15.89017767 | 9.434766667 | 19.96907667 | 11.030194 | 13.18859867 | 13.38303733 | 17.89872067 | 16.07230467 | 16.46560867 |
