# Supplementary material for: Deciphering intra-connectivity of gene network response to drought and salinity in apple
Source: Front Plant Sci. 2026 Mar 16;17:1763760. doi: 10.3389/fpls.2026.1763760 (PMC13033804; doi:10.3389/fpls.2026.1763760)
Supplement: Supplementary file 14 [file Table11.doc]

**Supplementary Table 11. Transcriptomic profiling of genes involved in Pyruvate metabolism**

| **Gene ID** | **Gene Name** | **Gene Annotation** | **CK_0** | **NaCl_1** | **NaCl_6** | **NaCl_12** | **NaCl_24** | **PEG_1** | **PEG_6** | **PEG_12** | **PEG_24** |
| --- | --- | --- | --- | --- | --- | --- | --- | --- | --- | --- | --- |
| MD00G1032700 | MdLLP | lactoylglutathione lyase family protein / glyoxalase I family protein | 8.264438 | 7.717248667 | 5.602836 | 3.984927333 | 4.764913333 | 6.990771667 | 6.608799 | 5.677693 | 5.896738 |
| MD01G1171200 | MdGLY2 | Metallo-hydrolase/oxidoreductase superfamily protein | 11.14331367 | 10.33204133 | 21.29113333 | 22.04126633 | 25.04031167 | 9.242235667 | 15.752868 | 17.63406033 | 24.57866667 |
| MD04G1093600 | MdACY | acylphosphatase family | 13.58200533 | 7.022646667 | 4.21539 | 1.872528333 | 2.537198333 | 7.595244333 | 5.466138333 | 6.183203667 | 3.571525333 |
| MD06G1177200 | MdALDH2B4 | aldehyde dehydrogenase 2B4 | 75.39529667 | 47.24367133 | 28.56964033 | 49.62462833 | 119.7626267 | 47.15085 | 51.340027 | 49.25412133 | 71.30821967 |
| MD08G1178000 | MdACC1 | acetyl-CoA carboxylase 1 | 32.453212 | 27.70901867 | 26.038945 | 12.62289467 | 10.94519667 | 51.319401 | 23.863608 | 26.13338067 | 14.631889 |
| MD09G1000400 | MdACS | acetyl-CoA synthetase | 28.08404667 | 29.427173 | 31.12205 | 60.65166467 | 55.54406867 | 31.55217567 | 44.79699033 | 29.87906767 | 30.32337567 |
| MD13G1177400 | MdPPDK | pyruvate orthophosphate dikinase | 0.011993667 | 0.02481 | 0.015499667 | 0.039258333 | 0.088649333 | 0.019891333 | 0.016235333 | 0 | 0.021616333 |
| MD16G1010900 | MdLMD | Lactate/malate dehydrogenase family protein | 3.724418333 | 5.701775667 | 8.614283 | 21.86780733 | 6.761283 | 4.994028667 | 17.042496 | 6.454546333 | 3.822437667 |
| MD16G1044200 | MdLTA2 | 2-oxoacid dehydrogenases acyltransferase family protein | 26.45023667 | 30.68993467 | 18.35065 | 7.855768667 | 7.632529333 | 30.97847933 | 24.05050733 | 19.18525633 | 9.833270667 |
| MD16G1145800 | MdLPD2 | lipoamide dehydrogenase 2 | 47.45283867 | 33.88409267 | 33.426722 | 30.71615167 | 34.10813267 | 33.66226967 | 36.46025133 | 42.53055333 | 33.362125 |
| MD16G1202600 | MdPKP-ALPHA | ADP-ribosylation factor C1 | 32.34759633 | 42.43512867 | 35.20113633 | 26.12560667 | 25.48803833 | 49.319701 | 34.91705567 | 30.19763433 | 20.889025 |
| MD17G1117800 | MdTFP | Thiolase family protein | 40.67331667 | 93.075915 | 112.2812627 | 139.191551 | 84.36410267 | 58.94315333 | 91.036827 | 71.96551267 | 68.617536 |
