# Supplementary material for: Deciphering intra-connectivity of gene network response to drought and salinity in apple
Source: Front Plant Sci. 2026 Mar 16;17:1763760. doi: 10.3389/fpls.2026.1763760 (PMC13033804; doi:10.3389/fpls.2026.1763760)
Supplement: Supplementary file 15 [file Table12.doc]

**Supplementary Table 12. Transcriptomic profiling of genes involved in porphyrin and chlorophyll metabolism**

| **Gene ID** | **Gene Name** | **Gene Annotation** | **CK_0** | **NaCl_1** | **NaCl_6** | **NaCl_12** | **NaCl_24** | **PEG_1** | **PEG_6** | **PEG_12** | **PEG_24** |
| --- | --- | --- | --- | --- | --- | --- | --- | --- | --- | --- | --- |
| MD15G1283600 | *MdACD2* | accelerated cell death 2 (ACD2) | 36.36479 | 13.67783667 | 21.509442 | 15.821093 | 17.30868367 | 14.38572967 | 20.25144333 | 18.24878667 | 15.405586 |
| MD15G1184100 | *MdNADP-1* | NAD(P)-binding Rossmann-fold superfamily protein | 5.497048 | 2.252483667 | 1.571408333 | 1.087886333 | 1.339611667 | 2.825788667 | 1.367277333 | 2.048044333 | 1.515583 |
| MD13G1227100 | *MdF420-HDRB* | coenzyme F420 hydrogenase family | 11.064848 | 7.086878667 | 5.077215333 | 2.457234333 | 4.016760333 | 6.883071333 | 6.154733 | 6.221438333 | 5.504256333 |
| MD13G1173600 | *MdNADP-2* | NAD(P)-binding Rossmann-fold superfamily protein | 27.78756967 | 9.7709 | 10.72143533 | 6.936358 | 6.177262 | 10.14003 | 13.00285533 | 13.56884467 | 8.934051667 |
| MD12G1256700 | *MdFC2* | ferrochelatase 2 | 24.46284767 | 9.199944667 | 14.46877167 | 15.01046767 | 12.363196 | 8.790759 | 16.783219 | 18.46692967 | 20.73999367 |
| MD11G1187400 | *MdALB1* | ALBINA 1 | 0 | 0 | 0 | 0 | 0.124432333 | 0.043383 | 0.081321667 | 0.043055667 | 0.128179667 |
| MD11G1149200 | *MdCH1-1* | Pheophorbide a oxygenase family protein with Rieske [2Fe-2S] domain | 18.973366 | 8.324000333 | 6.360831333 | 6.668274333 | 15.03953367 | 8.640545 | 7.746161333 | 8.750587667 | 11.296766 |
| MD09G1291200 | *MdPPOX* | Flavin containing amine oxidoreductase family | 29.82482767 | 29.08880467 | 17.52719767 | 9.755113333 | 10.614159 | 28.045819 | 20.25976067 | 16.36223333 | 15.36191433 |
| MD09G1118200 | *MdGUN5* | Mg-protoporphyrin IX chelatase, putative (CHLH) | 0.054229667 | 0.074367 | 0.142860667 | 0.010816 | 0.066160667 | 0.086847333 | 0.076972333 | 0.079729333 | 0.118295667 |
| MD08G1162200 | *MdCH1-2* | Pheophorbide a oxygenase family protein with Rieske [2Fe-2S] domain | 15.420714 | 3.348499667 | 8.354676 | 9.863116 | 10.71583433 | 4.8089 | 7.200325333 | 20.58760333 | 9.72395 |
| MD07G1002800 | *MdUPM1* | urophorphyrin methylase 1 | 8.457083667 | 227.183192 | 88.247893 | 75.39770767 | 102.5481987 | 109.166962 | 87.617639 | 22.69663033 | 34.83466267 |
| MD06G1131500 | *MdCHLM* | magnesium-protoporphyrin IX methyltransferase | 68.72927233 | 18.66354267 | 28.47289267 | 17.335518 | 21.67404333 | 19.06932767 | 40.20219833 | 34.70193533 | 25.33090533 |
| MD05G1304900 | *MdPLKN* | P-loop containing nucleoside triphosphate hydrolases superfamily protein | 21.65497967 | 13.06299633 | 14.479931 | 10.505776 | 11.312493 | 12.772802 | 14.98663133 | 14.33768567 | 12.18371167 |
| MD03G1259000 | *MdCLH1* | chlorophyllase 1 | 4.407656333 | 23.11946567 | 8.634770333 | 2.601052 | 1.497700667 | 16.454168 | 5.395721333 | 4.791670333 | 3.602481667 |
| MD03G1223800 | *MdPORA* | protochlorophyllide oxidoreductase A | 302.6492157 | 101.166761 | 194.609019 | 97.195788 | 124.8149717 | 114.3521983 | 221.521469 | 241.1544343 | 197.4644473 |
| MD00G1131800 | *MdNYE1* | non-yellowing 1 | 1.211605667 | 0.861297667 | 0.769862 | 0.989616333 | 1.244371667 | 1.126102 | 1.020043667 | 2.166002333 | 1.406240667 |
| MD00G1107700 | *MdCRD1* | dicarboxylate diiron protein, putative (Crd1) | 1474.556254 | 565.029978 | 622.177836 | 711.3403323 | 857.3920897 | 566.4701947 | 792.6218463 | 1081.670288 | 788.6316933 |
