# Supplementary material for: Deciphering intra-connectivity of gene network response to drought and salinity in apple
Source: Front Plant Sci. 2026 Mar 16;17:1763760. doi: 10.3389/fpls.2026.1763760 (PMC13033804; doi:10.3389/fpls.2026.1763760)
Supplement: Supplementary file 16 [file Table13.doc]

**Supplementary Table 13. Transcriptomic profiling of genes involved in carbon fixation in photosynthetic organisms**

| **Gene ID** | **Gene Name** | **Gene Annotation** | **CK_0** | **NaCl_1** | **NaCl_6** | **NaCl_12** | **NaCl_24** | **PEG_1** | **PEG_6** | **PEG_12** | **PEG_24** |
| --- | --- | --- | --- | --- | --- | --- | --- | --- | --- | --- | --- |
| MD04G1222500 | *MdRPIA* | Ribose 5-phosphate isomerase, type A protein | 59.57019933 | 45.26543967 | 16.04102967 | 10.88067867 | 15.18134767 | 40.88487633 | 28.24361167 | 21.23756433 | 10.815766 |
| MD07G1119500 | *MdTSK* | Transketolase | 240.801122 | 191.4321697 | 150.1643473 | 161.4880167 | 169.4600373 | 191.437276 | 194.8688813 | 150.7714157 | 136.731705 |
| MD13G1102800 | *MdAOAT2* | alanine-2-oxoglutarate aminotransferase 2 | 229.374842 | 84.26958467 | 50.55373033 | 64.54895767 | 60.77081267 | 73.876363 | 87.249013 | 81.290553 | 52.69249967 |
| MD17G1003500 | *MdGAPA-2* | glyceraldehyde 3-phosphate dehydrogenase A subunit 2 | 458.566386 | 198.7455853 | 100.7734147 | 48.63767767 | 50.148037 | 170.088959 | 157.8606667 | 123.5420023 | 70.71386433 |
| MD17G1014000 | *MdADT* | Aldolase-type TIM barrel family protein | 3.673669333 | 3.246922333 | 4.930482 | 6.899297667 | 5.654813 | 3.120709333 | 3.862023 | 5.232254667 | 5.134414333 |
