# Supplementary material for: Deciphering intra-connectivity of gene network response to drought and salinity in apple
Source: Front Plant Sci. 2026 Mar 16;17:1763760. doi: 10.3389/fpls.2026.1763760 (PMC13033804; doi:10.3389/fpls.2026.1763760)
Supplement: Supplementary file 17 [file Table14.doc]

**Supplementary Table 14. Transcriptomic profiling of genes involved in valine, leucine and isoleucine degradation**

| **Gene ID** | **Gene Name** | **Gene Annotation** | **CK_0** | **NaCl_1** | **NaCl_6** | **NaCl_12** | **NaCl_24** | **PEG_1** | **PEG_6** | **PEG_12** | **PEG_24** |
| --- | --- | --- | --- | --- | --- | --- | --- | --- | --- | --- | --- |
| MD11G1280500 | *MdMCCA* | methylcrotonyl-CoA carboxylase alpha chain | 18.58081533 | 7.700034 | 44.60845267 | 89.15359767 | 69.82559967 | 8.813914 | 28.73746433 | 38.90517133 | 85.25968933 |
| MD07G1111300 | *MdCLP* | ATP-dependent caseinolytic (Clp) protease/crotonase family protein | 5.309098667 | 4.730369333 | 5.313534 | 4.027185667 | 4.653970333 | 4.889011 | 5.139064333 | 5.392637667 | 4.984204333 |
| MD05G1082300 | *MdMCCB* | 3-methylcrotonyl-CoA carboxylase | 40.319908 | 27.33203067 | 78.91139367 | 137.1389873 | 117.118561 | 27.14899633 | 63.48946767 | 76.38642 | 114.7476093 |
| MD03G1116100 | *MdIVD* | isovaleryl-CoA-dehydrogenase | 22.41620033 | 7.702786333 | 26.58664067 | 74.33773933 | 68.43670667 | 7.920213 | 22.71388533 | 34.72155167 | 59.13991433 |
| MD03G1002200 | *MdBCDH* | branched-chain alpha-keto acid decarboxylase E1 beta subunit | 7.343143 | 3.712354667 | 12.81098 | 20.982583 | 21.113603 | 5.544589333 | 10.88843433 | 13.21857767 | 23.08783667 |
